# Intraosseous tumours of the knee

**DOI:** 10.1007/s00256-025-05105-y

**Published:** 2026-01-20

**Authors:** Luqman Wali, Ganesh Hegde, Michael Khoo, Ian Pressney, Lizz van der Heijden, Michael Bus, Roberto Tirabosco, Bhavin Upadhyay, Paul O’Donnell

**Affiliations:** 1 Clinical Radiology, Royal National Orthopaedic Hospital, Stanmore, UK; 2 Institute of Orthopaedics and Musculoskeletal Science, University College, London, UK; 3 Sarcoma and Joint Reconstruction Unit, Royal National Orthopaedic Hospital, Stanmore, UK; 4 Clinical Histopathology, Royal National Orthopaedic Hospital, Stanmore, UK; 5 Research Department of Pathology, UCL Cancer Institute, London, UK

**Keywords:** Sarcoma, Tumour, Knee, Subarticular, Metaphyseal, Non-neoplastic, Benign, Intermediate, Malignant

## Abstract

Neoplastic and non-neoplastic lesions occur commonly in the bones of the knee. For certain tumours, it is the most frequent location, and it is also a common site for neoplasms and non-neoplastic tumour mimics that do not demonstrate a particular anatomic preference. Lesions may arise at the end of the bone (subarticular) or in the metaphysis and extend towards the joint. Tumours and tumour mimics occurring in subarticular locations or extending from the metaphysis are considered separately in this review. We have included lesions that we expect will be identifiable on routine knee MRI examinations.

**Supplementary Information:**

The online version contains supplementary material available at 10.1007/s00256-025-05105-y.

## Introduction

The knee is a common site for intraosseous primary tumours and non-neoplastic tumour mimics. The distal femur and proximal tibia are the commonest locations for giant cell tumour of bone; conventional osteosarcoma most commonly occurs in the distal femoral metaphysis and involvement of the knee joint is frequent; lesions that occur in epiphyses or apophyses are also found in sesamoid bones such as the patella. Parosteal osteosarcoma, the commonest surface bone-forming malignancy, occurs at the posterior aspect of the distal femur in 70% of cases and may present with restricted knee flexion. Bone tumours that do not demonstrate a particular anatomic preference also commonly occur at the knee. Non-neoplastic tumour mimics, such as cysts, osteonecrosis, subarticular fractures and developmental variants, are frequent.

In this review, we will consider intraosseous lesions affecting the end of the bone (subarticular) and metaphyseal lesions that extend to the end of the bone and which may be identified on routine knee MRI examinations. A summary of these conditions, with salient imaging features, is given in Table 1 in the Appendix.

## Neoplastic lesions

### Subarticular

#### Giant cell tumour (GCT)

GCT is a locally aggressive bone tumour, occasionally metastasising to lung (Fig. S1), accounting for approximately 4–10% of all primary bone tumours and 20% of benign bone tumours. Patients are usually 20–40 years old (although GCT can occur in children), and there is a slight female predominance (1.5:1) [1]. An *H3F3A* gene mutation, usually involving p.Gly34Trp (p.G34W), has been detected in > 95% of GCT [2].

In long bones, the tumour occurs in the epiphysis, with the distal femur, proximal tibia and distal radius being most frequent [3, 4]. It also occurs in apophyses and sesamoids, which at the knee include the patella [5].

Eccentric subarticular bone lysis with cortical destruction and the appearance of an extraosseous mass (usually contained by periosteum) are typical findings. The bone margins are not sclerotic, frequently ill-defined, and there is usually no significant internal matrix mineralisation or reactive periostitis [2, 6].

**Fig. 1:**
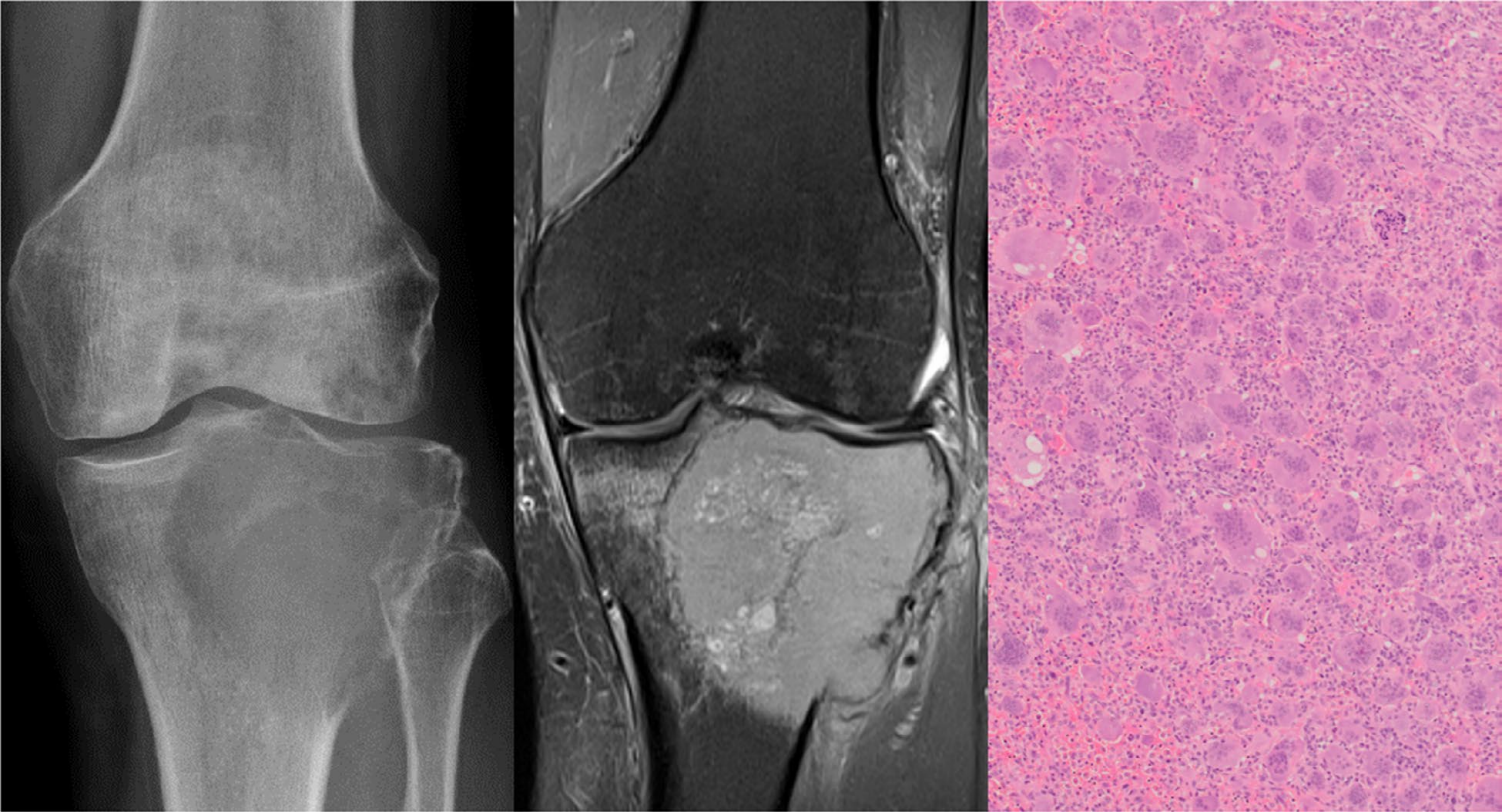
Fifty-three-year-old male with tibial giant cell tumour. AP knee radiograph (left) reveals a lytic subarticular proximal tibial lesion without a clear sclerotic margin. Associated cortical attenuation with regions of cortical deficiency. Coronal PDFS image (centre) confirms a subarticular expansile lesion with focal cortical breach and adjacent oedema. Typical features of a giant cell tumour of bone show mononuclear stromal cells with numerous intervening osteoclast-like giant cells (right) (4 × magnification. H&E stain)

**Fig. 2:**
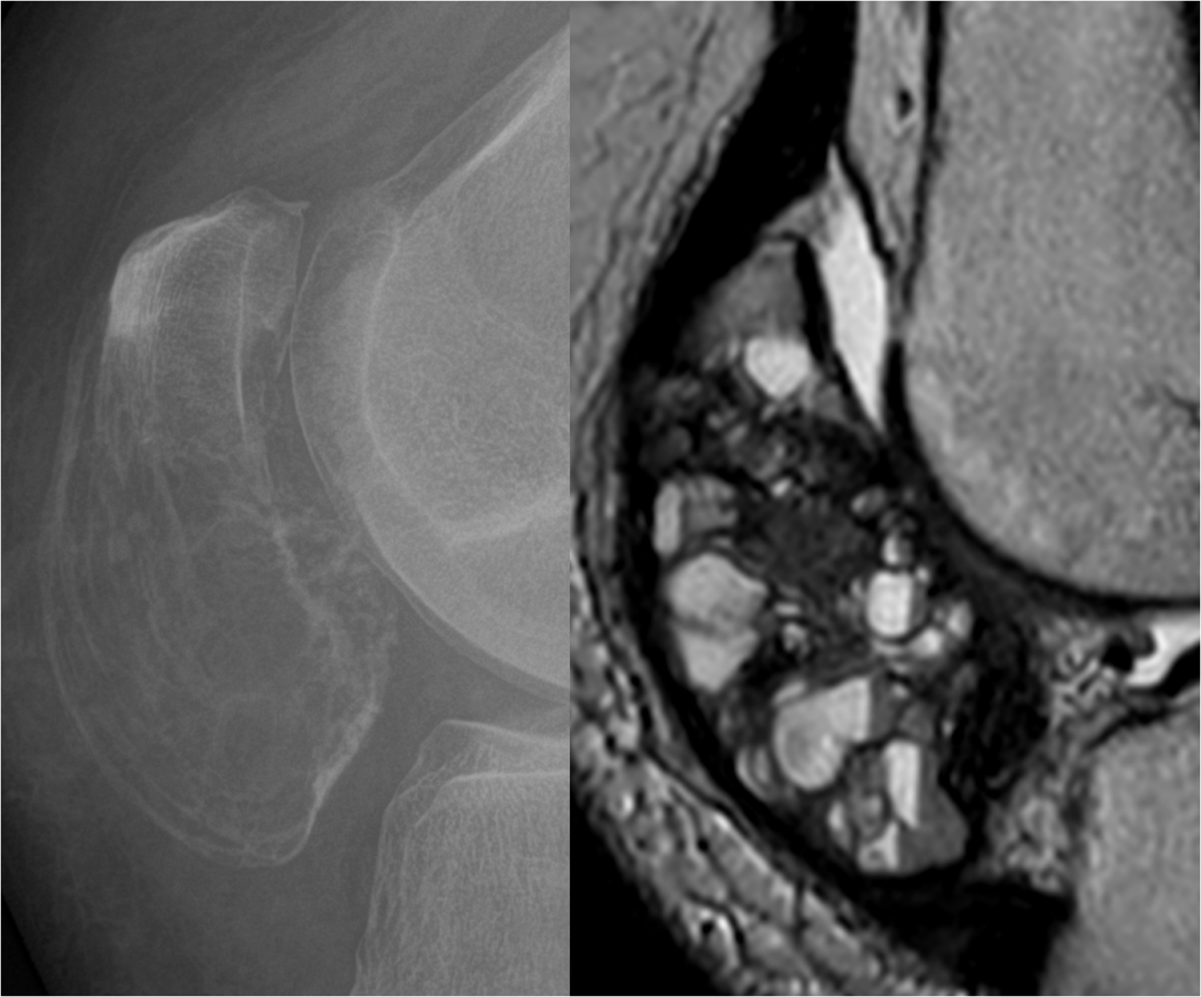
Fifty-five-year-old female with patellar giant cell tumour. Lateral knee radiograph (left) demonstrates a markedly expansile lytic patellar lesion with prominent associated cortical attenuation and effacement of the infrapatellar fat pad. Sagittal T2-weighted image (right) shows ABC-like changes with multiple fluid–fluid levels

Useful diagnostic magnetic resonance (MR) features of GCT include intermediate to low signal on T1-weighted imaging and intermediate to high signal on T2-weighted imaging (WI), with accentuated T2* gradient echo (GE) hypointensity, highlighting haemosiderin deposition from chronic haemorrhage [6, 7] (Fig. 1). Ten to fifteen percent of GCT’s will demonstrate multiple fluid–fluid levels suggesting aneurysmal bone cyst-like change [4, 8] (Fig. 2).

**Fig. 3:**
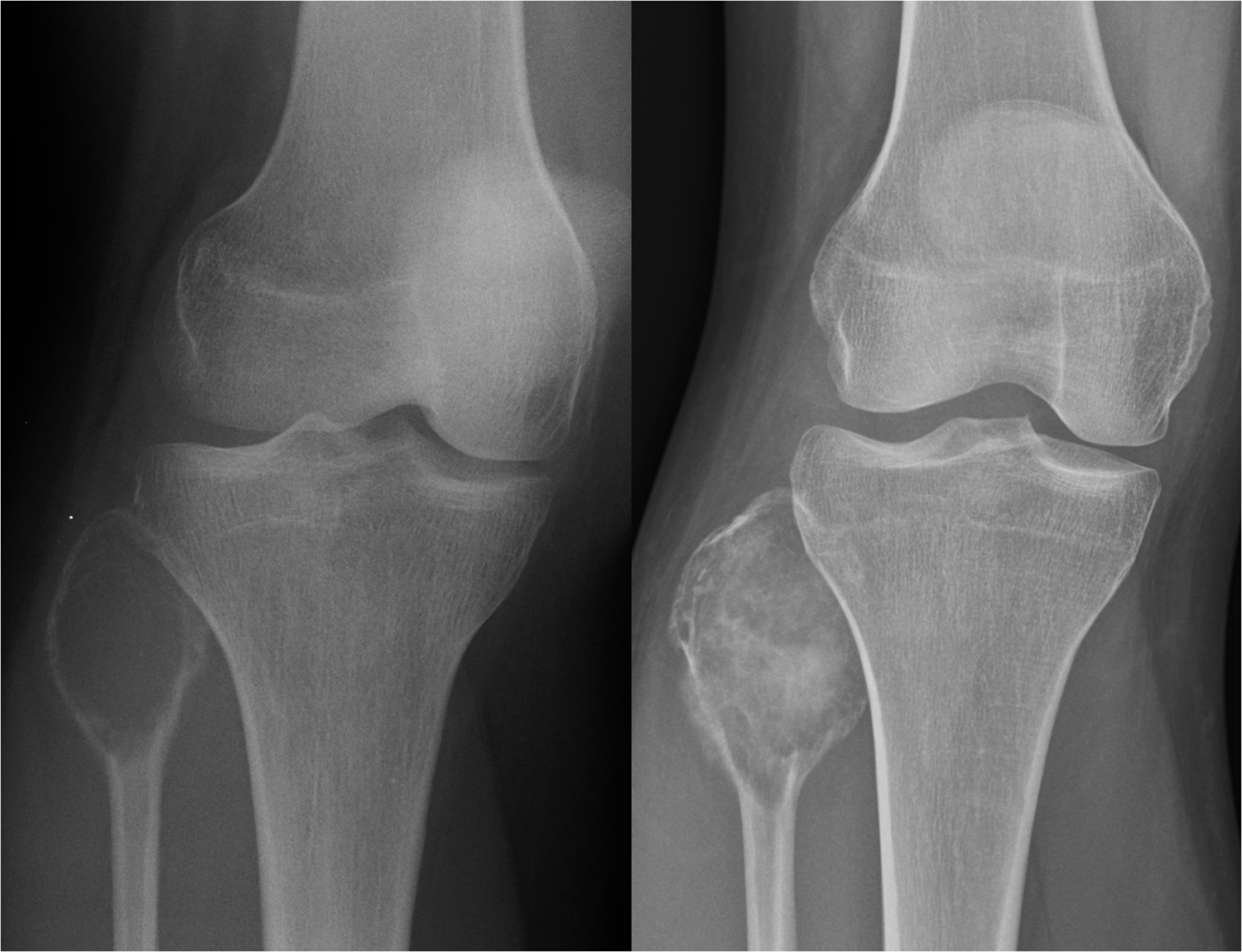
Fifty-five-year-old female with proximal fibular giant cell tumour and lung metastases (see Fig. S1). AP knee radiograph (left) reveals a markedly expansile lytic proximal fibular lesion with marked associated cortical attenuation. Thirty-seven-year-old female with proximal fibular malignant giant cell tumour with osteosarcomatous differentiation. AP knee radiograph (right) reveals an expansile proximal fibular lesion with sclerosis suggestive of matrix mineralisation (in keeping with the known osteosarcomatous differentiation) and a small ossified extraosseous mass laterally

On rare occasions, GCT can show malignant histological features, either de novo or following surgery or radiotherapy (Fig. 3) [6].

Extensive intralesional curettage is the preferred treatment of low-risk GCT (confined to bone, with minimal extraosseous extension and/or simple fracture) and is typically combined with local adjuvants, such as high-speed burring, phenol, polymethylmethacrylate (PMMA) cement to reduce local recurrence [9–11]. En bloc resection and endoprosthetic replacement are performed in high-risk GCT (with complex fractures, extensive extraosseous involvement or recurrent tumours) [3]. Denosumab reduces osteoclast activity and promotes bone formation, but does not induce stromal cell apoptosis [12]. Bisphosphonates can also be used to reduce bone resorption and stabilise disease [13]. Both are options for unresectable GCT and can help facilitate surgery.

#### Chondroblastoma (CB)

CB is a benign cartilaginous neoplasm representing 1% of all bone tumours. It occurs predominantly in young adults but affects a wide age range (10–60 years) and shows a 2:1 male predominance [14, 15].

Typically affecting the epiphysis, it can also arise in an apophysis or sesamoid bone such as the patella: lesions can involve the metaphysis even if the physis is open [16–18]. Approximately 75% arise in the knee, hip and shoulder [19]; 2% occur in the diametaphyses of long bones [20].

CBs produce prostaglandins, causing reactive bone marrow oedema and periostitis, as well as effusion and synovitis in the adjacent joint. Clinical features such as pain, swelling and restricted movement can mimic other pathologies, such as a meniscal tear, infection or inflammatory arthropathy [19, 21, 22].

**Fig. 4:**
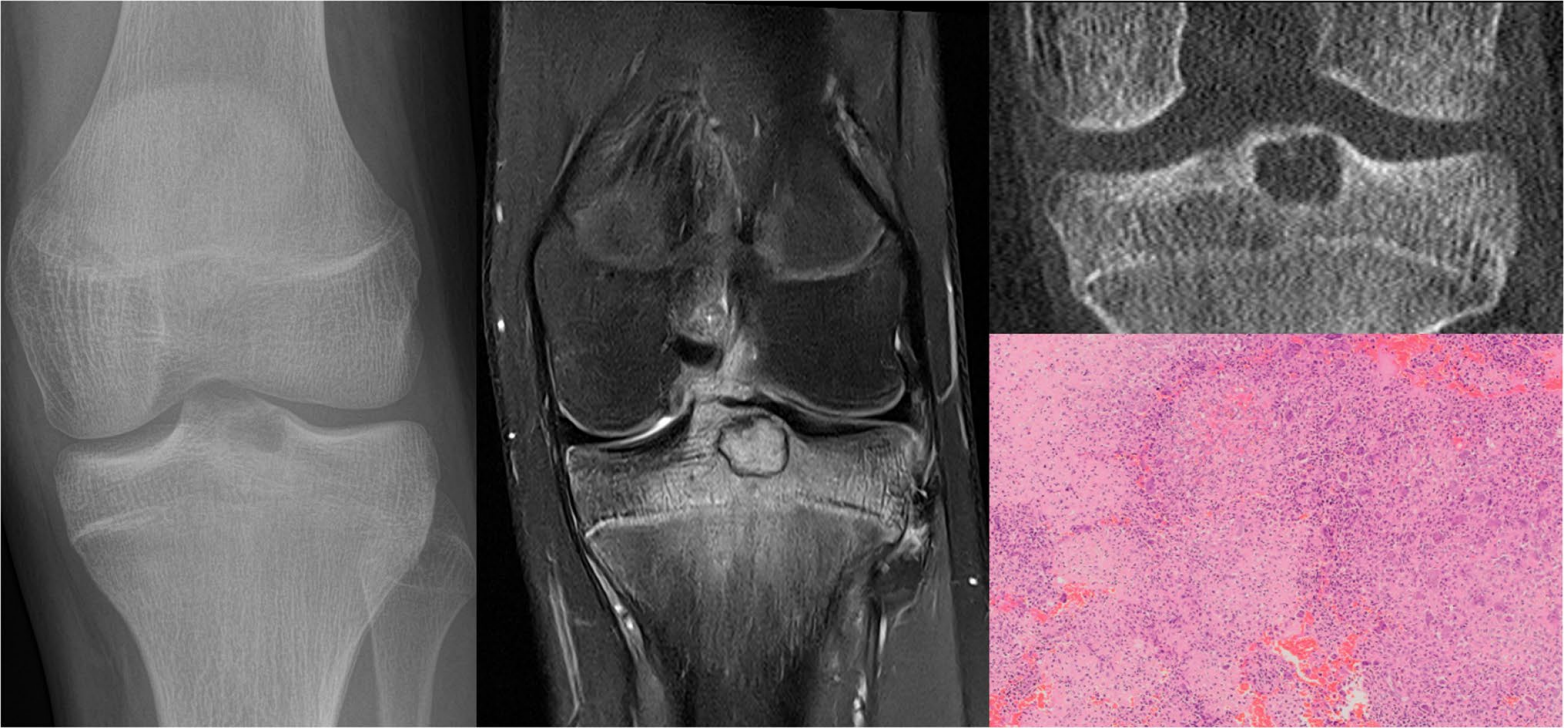
Seventeen-year-old male with proximal tibial epiphyseal chondroblastoma. AP knee radiograph (left) shows a well-defined lytic lesion within the intercondylar eminence. Coronal STIR image (centre) shows a corresponding lobular lesion with a well-defined peripheral low signal margin and marked perilesional oedema. Coronal reconstruction of the CT-guided biopsy planning study (top right) reveals subtle intralesional chondroid matrix mineralisation, which is difficult to appreciate radiographically. Sheets of mononuclear stromal cells with scattered osteoclast-like giant cells admixed with chondroid lobules (bottom right) (4 × magnification, H&E stain)

Radiographs will demonstrate a well-defined, lucent and lobulated epiphyseal lesion with a sclerotic margin, with up to half demonstrating flocculant or punctate internal calcification, best demonstrated by computed tomography (CT) [14, 23] (Fig. 4). A solid periosteal reaction is commonly seen in long bone cases of giant cell tumour, occurring in about 57% of instances, though multi-lamellated patterns may also be observed. In subarticular lesions, periosteal reaction is limited to the metadiaphyseal cortex, as the epiphysis does not support periosteal bone formation [24].

**Fig. 5:**
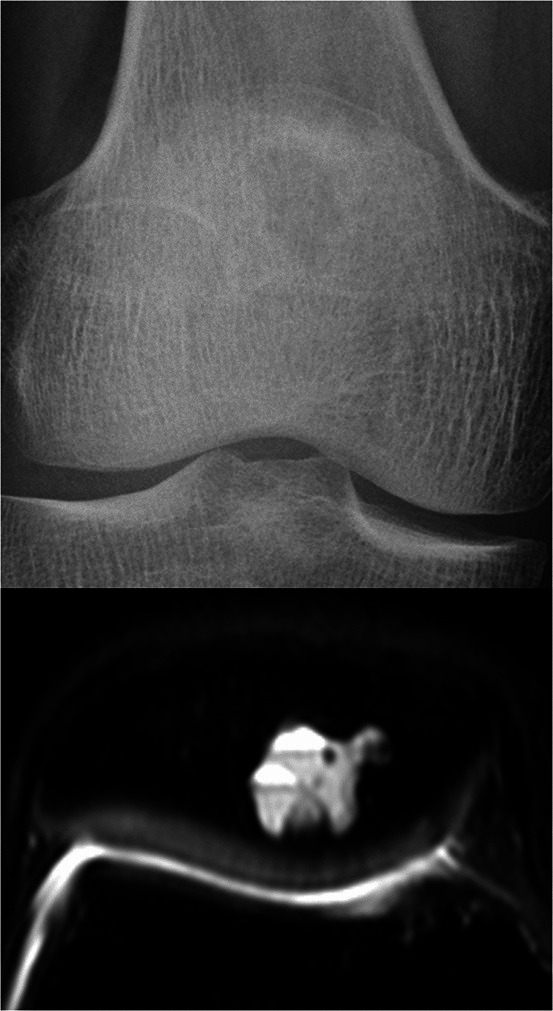
Forty-four-year-old male with patellar chondroblastoma. AP knee radiograph (top) identifies a well-defined lytic lesion within the patella with a well-defined sclerotic margin. Intralesional chondroid matrix mineralisation is difficult to appreciate. Axial PDFS image (bottom) reveals a corresponding lobular lesion with internal fluid–fluid levels. Despite the lack of perilesional oedema, the location suggests ABC-like changes within a chondroblastoma. An epiphyseal lesion containing multiple fluid–fluid levels suggests a pre-existing chondroblastoma or giant cell tumour even without a clear solid component

Heterogeneous but usually hypointense signal on T1WI and fluid-sensitive MRI sequences is seen, with further low signal foci reflecting calcification [25]. The reactive changes (bone and soft tissue oedema, periosteal reaction and effusion/synovitis) are other typical MRI findings but are occasionally absent (Fig. 5). MRI can also demonstrate multiple fluid–fluid levels suggesting secondary ABC-like change (demonstrated in 15–20% of lesions) [19, 23] (Fig. 5).

Surgical management consists of intralesional curettage with local adjuvants (bone cement, phenol, hydrogen peroxide or cryotherapy [16, 26]). In young patients, the cavity is usually filled with bone graft. Smaller lesions can be targeted with radiofrequency ablation, with en bloc resection rarely needed for aggressive cases. Local recurrence varies from 10 to 35%, depending on location and technique utilised [17, 27, 28].

#### Osteoid osteoma (OO)

OO is a common benign bone-forming tumour, accounting for approximately 10% of all primary bone tumours [29, 30]. There is a male predominance (3:1), with the tumour typically seen in adolescents and young adults [31]. The tumour produces prostaglandins, causing surrounding reactive changes (similar to CB) and pain, usually disproportionate to the size of the lesion, classically worse at night and relieved by non-steroidal anti-inflammatory drugs (NSAIDs) [32].

OO is most common in the diaphysis of long bones, accounting for over 50% of all cases [33]. Most are intracortical (75%), less commonly intramedullary and rarely subperiosteal [34, 35].

**Fig. 6:**
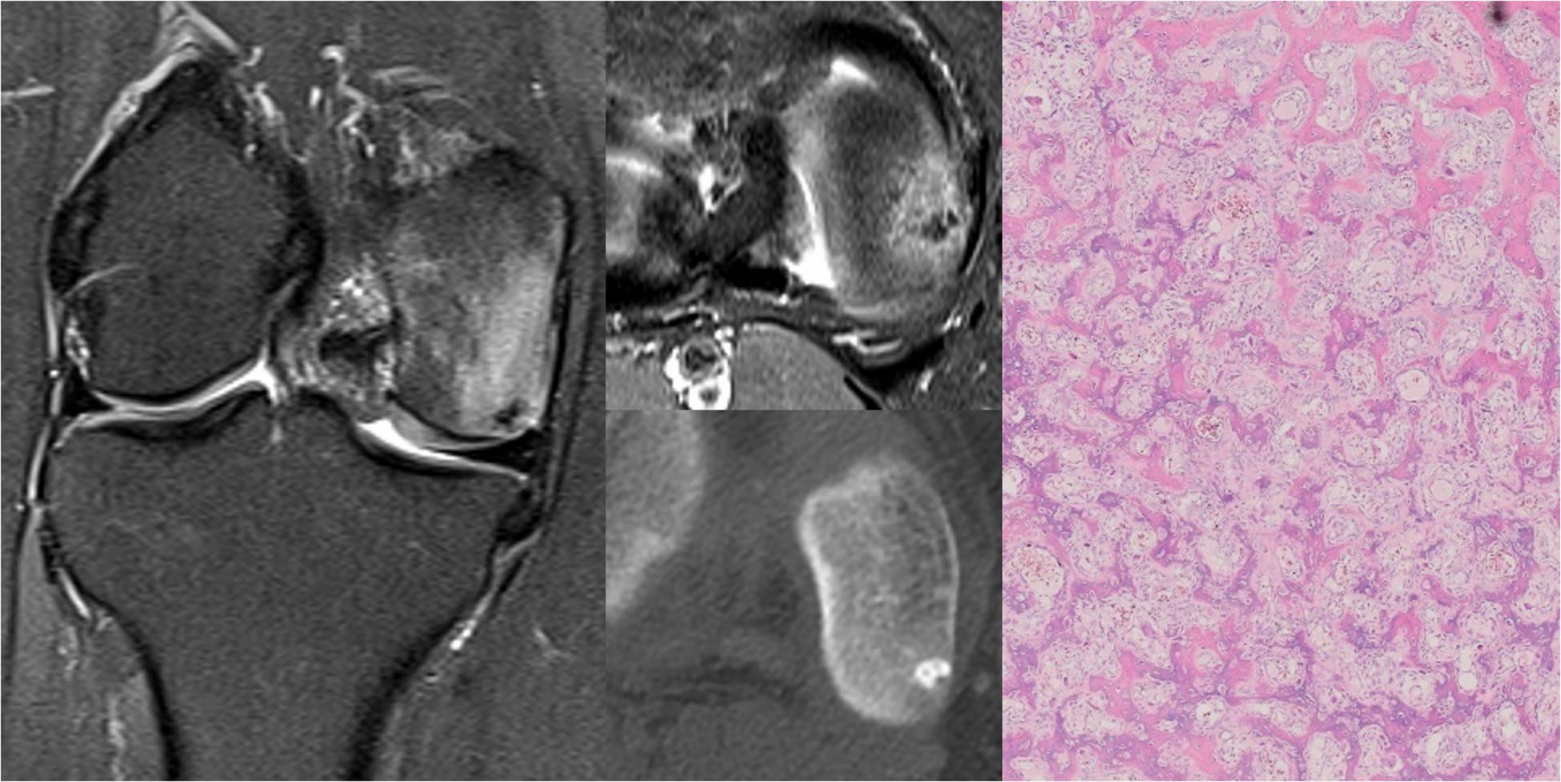
Twenty-four-year-old female with osteoid osteoma. Coronal STIR (left) and axial PDFS (top centre) images reveal focal low signal within the medial femoral condyle with prominent corresponding oedema-like marrow signal. Axial CT reconstruction (bottom centre) image better depicts the subchondral sclerotic nidus. Anastomosing trabeculae of woven bone lined by plump osteoblasts with an intervening fibrovascular stroma (right) (4 × magnification. H&E stain)

OO usually consists of a small (< 2 cm), well-defined, bone-forming tumour (the nidus), which shows variable central mineralisation. It is surrounded by sclerosis, with MRI showing peritumoral oedema (Fig. 6). Imaging features are determined by location: cortical long bone OO causes fusiform, asymmetric cortical thickening within which resides a small lucent nidus [31]. CT is frequently needed to demonstrate the nidus and shows intracortical lucent vascular channels (the vascular groove sign) [36]. MRI will demonstrate the reactive bony changes: the nidus is frequently seen but may be too small for MRI detection [37].

Intra-articular OO mimics an arthropathy clinically and radiologically, and, as a result, the diagnosis is frequently delayed [31, 38]. Patients present with joint swelling, reduced range of motion, an effusion and synovitis, whilst nocturnal pain and the response to salicylates are less marked [39]. MRI shows synovial changes and marrow oedema, but typically, CT is required to demonstrate the nidus (Fig. 6).

Management is dependent on symptoms and location. A conservative approach should be considered with the use of non-steroidal anti-inflammatory drugs (NSAIDs): spontaneous regression may take 2–6 years [40]. Radiofrequency ablation (RFA) is a well-established minimally invasive technique [41]. If RFA is unsuccessful or deemed unsafe, curettage can be performed [37, 42].

## Metaphyseal

### Osteosarcoma (OS)

OS is a malignant mesenchymal tumour that produces neoplastic bone and can be classified by grade, site of origin (intramedullary or surface, the latter including parosteal, periosteal, intracortical and high-grade surface) and histological subtypes (the commonest being osteoblastic, chondroblastic and fibroblastic).

#### Conventional OS

Conventional OS (COS) is the most common subtype, defined as a high-grade, intramedullary bone-forming tumour. It shows a bimodal age distribution, most cases occurring in teenagers, but approximately 30% occur in patients over 40 years old [43–45]. The majority originate in the long bones, especially in the distal femur (30%) and proximal tibia (15%) [46]. The tumour is usually metaphyseal, arising in the epiphysis in < 2% of cases. However, involvement of the epiphysis by metaphyseal tumour is common [47, 48].

**Fig. 7:**
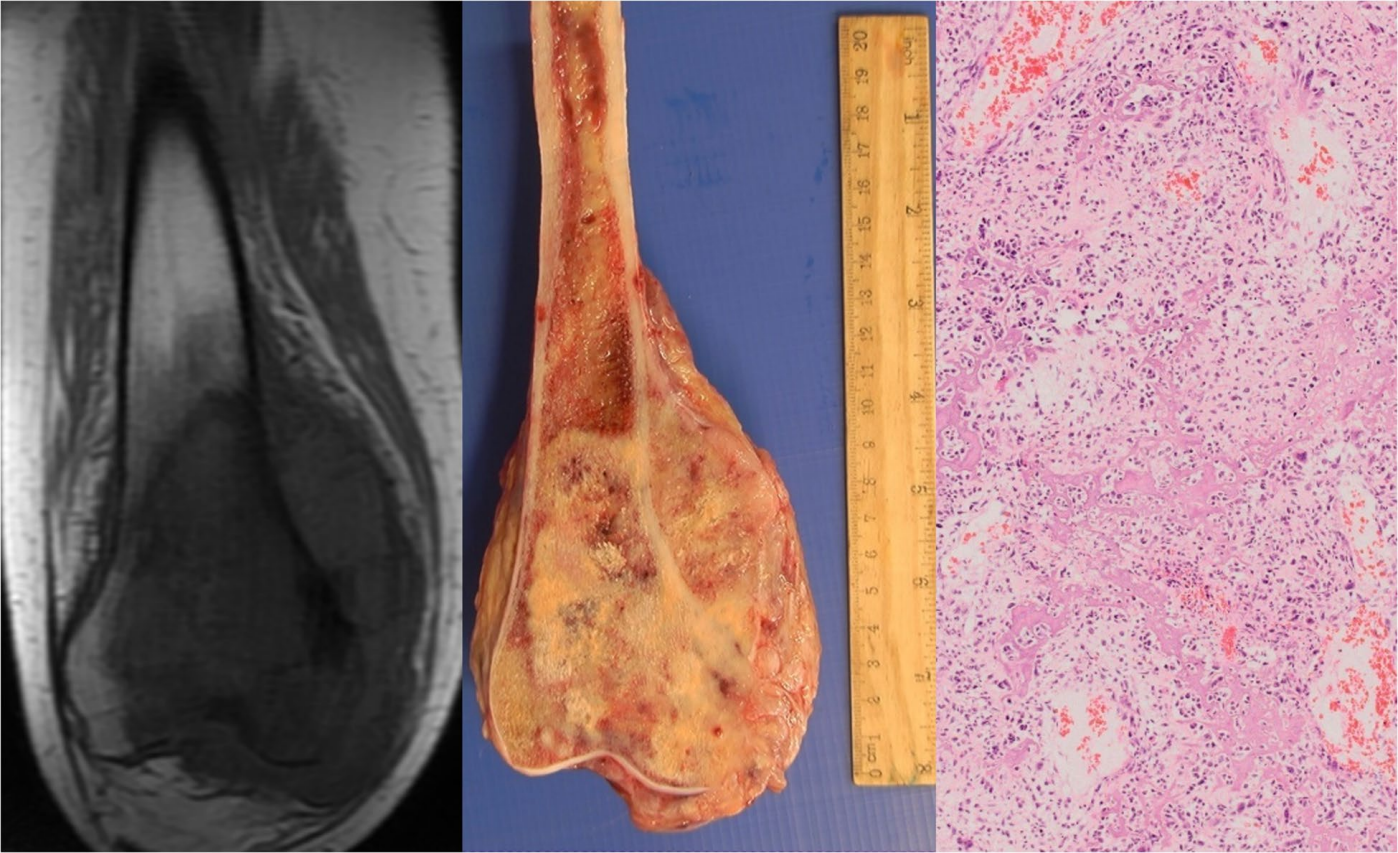
Sixteen-year-old female with conventional central osteosarcoma. Coronal T1-weighted image (left) shows distal femoral marrow infiltration with prominent extraosseous soft tissue, which elevates the periosteum and demonstrates intra-articular extension inferiorly. Areas of more prominent low signal suggestive of osteoid matrix mineralisation. Corresponding resection specimen (centre) confirms an ossified intramedullary distal femoral tumour with periosteal elevation, extraosseous and intra-articular extension. Severely atypical epithelioid cells with typical lace-like osteoid matrix (right) (4 × magnification; H&E stain)

Radiographs typically demonstrate an aggressive lytic/sclerotic lesion with a wide zone of transition and cloud-like mineralisation [49]. Cortical destruction with periosteal displacement and ossification causes radiographically visible ‘onion skin’ (multiple layers) and ‘sunburst’ (perpendicular) periosteal responses and frequently a Codman triangle [50, 51]. CT can identify radiographically occult mineralisation. The best modality for local staging is MRI, which assesses intramedullary (lesion length, epiphyseal involvement and skip metastases) and extraosseous (compartmental involvement, neurovascular encasement and joint extension) tumour accurately [52]. Typically, OS shows heterogenous intermediate signal on T1WI and hyperintensity on fluid-sensitive sequences mixed with hyperintense haemorrhagic and hypointense mineralised regions [49] (Fig. 7).

#### Parosteal OS

Parosteal OS (pOS) is the commonest surface OS. About 70% occur just superior to the knee at the posterior aspect of the distal femur. It arises from the outer layer of the periosteum, is usually a low-grade lesion and affects a slightly older population than COS [53, 54].

**Fig. 8:**
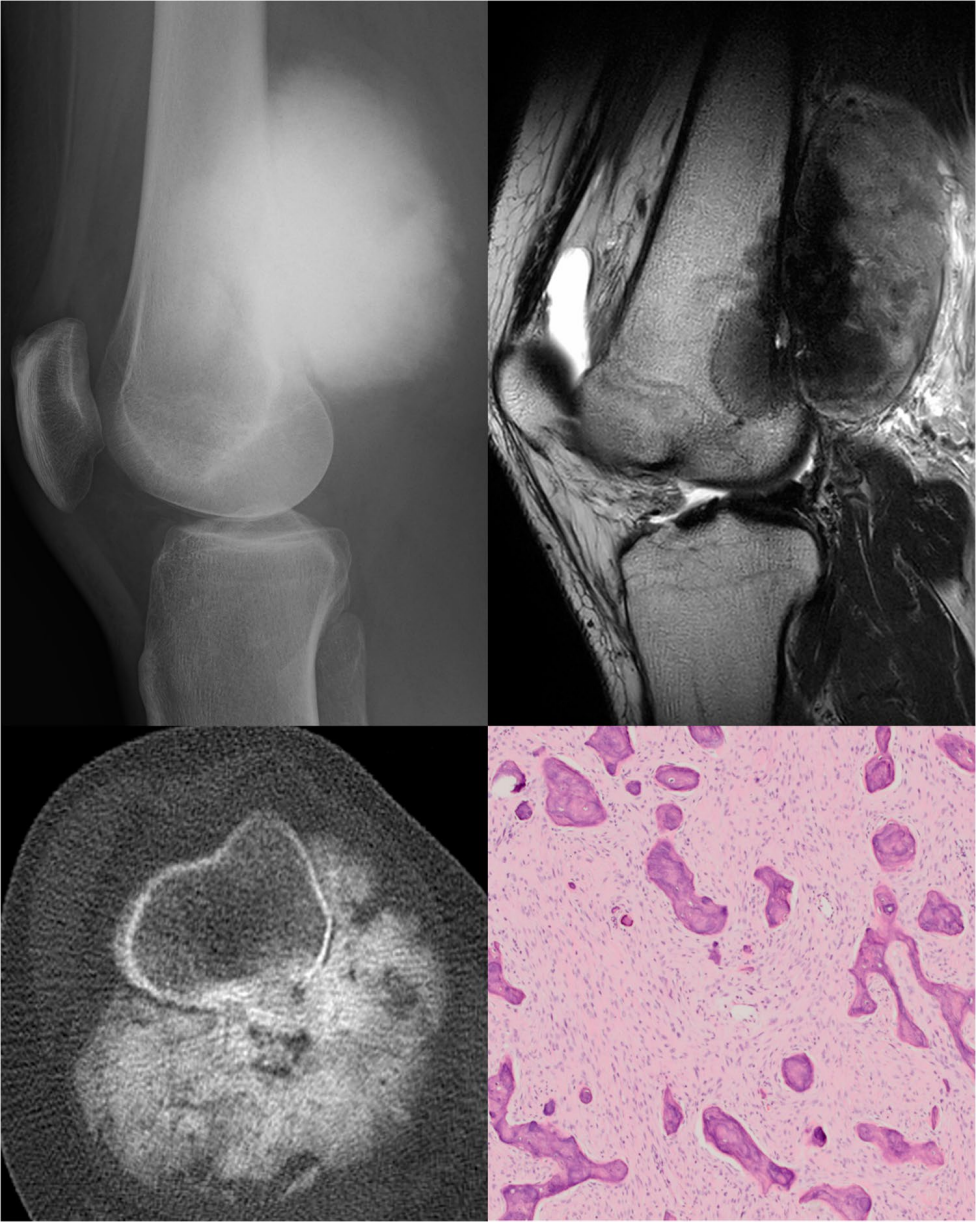
Twenty-four-year-old male with parosteal osteosarcoma. Lateral knee radiograph (top left) reveals a lobular heavily mineralised mass closely related to the posterior distal femoral metadiaphysis, Sagittal T2-weighted image (top right) demonstrates a hypointense mass with intramedullary extension. Axial CT image (bottom left) from the biopsy planning study better depicts the lucent cleavage plane related to the intervening intact periosteum interposed between the femur and the heavily mineralised tumour. Irregular trabeculae of woven bone embedded within a moderately cellular spindle cell component within a fibrous stroma (bottom right) (4 × magnification. H&E stain)

**Fig. 9:**
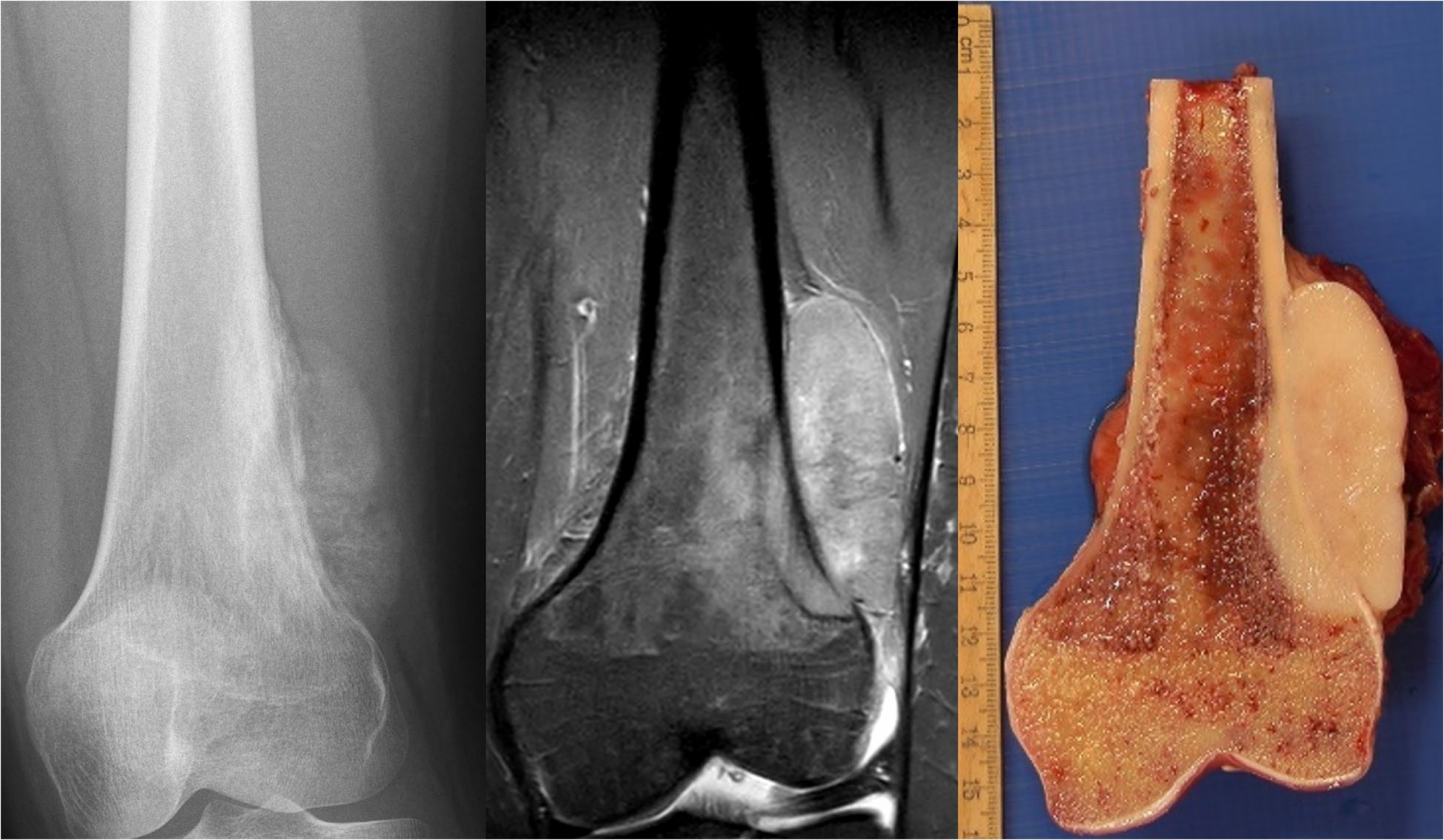
Thirty-year-old female with parosteal osteosarcoma. AP knee radiograph (left) reveals a lobular heavily mineralised mass closely related to the posterolateral distal femoral metadiaphysis. Coronal STIR image (centre) demonstrates a surface lesion with intramedullary extension. Corresponding resection specimen (right) confirms a homogeneous lobular ossified surface lesion with intramedullary extension

Radiographs usually show a large, lobular, ossified mass at the bone surface with the density greater centrally than peripherally (Fig. 8). Its attachment to the bone may be slender, with an interposed lucent plane thought to represent the intact periosteum. CT can demonstrate cortical thickening and sclerotic intramedullary extension (Figs. 8, 9). MRI typically demonstrates a hypointense, heterogenous mass, but the presence of hyperintensity on T2WI in regions of unossified tumour suggests high-grade transformation, usually also histologically OS [55].

Management of conventional osteosarcoma consists of neoadjuvant chemotherapy, surgery and adjuvant chemotherapy. En bloc surgical resection is the cornerstone of osteosarcoma treatment. Limb salvage techniques use endoprostheses, biological reconstructions or an allograft-prosthetic combination (APC); ablative techniques include amputation and rotationplasty. If the knee joint and adjacent epiphysis are not involved, knee sparing techniques can be used. Osteosarcomas are not radiation sensitive, and radiotherapy is usually palliative. Parosteal osteosarcomas are usually low grade and treated with wide surgical excision only [56].

### Chondrogenic tumours

Enchondromas (En) are benign intramedullary lesions, usually found incidentally [57] with an estimated population prevalence on knee MRI of 2.8% [57]. Atypical cartilaginous tumours (ACT) are locally aggressive chondral lesions in the appendicular skeleton, most commonly the distal femur. They are co-classified with the histologically identical grade 1 chondrosarcomas (CS1), which affect the axial skeleton and flat bones. Grades 2 and 3 CS are high-grade malignant tumours: chondromas and CS can be central/intramedullary or periosteal [58].

**Fig. 10:**
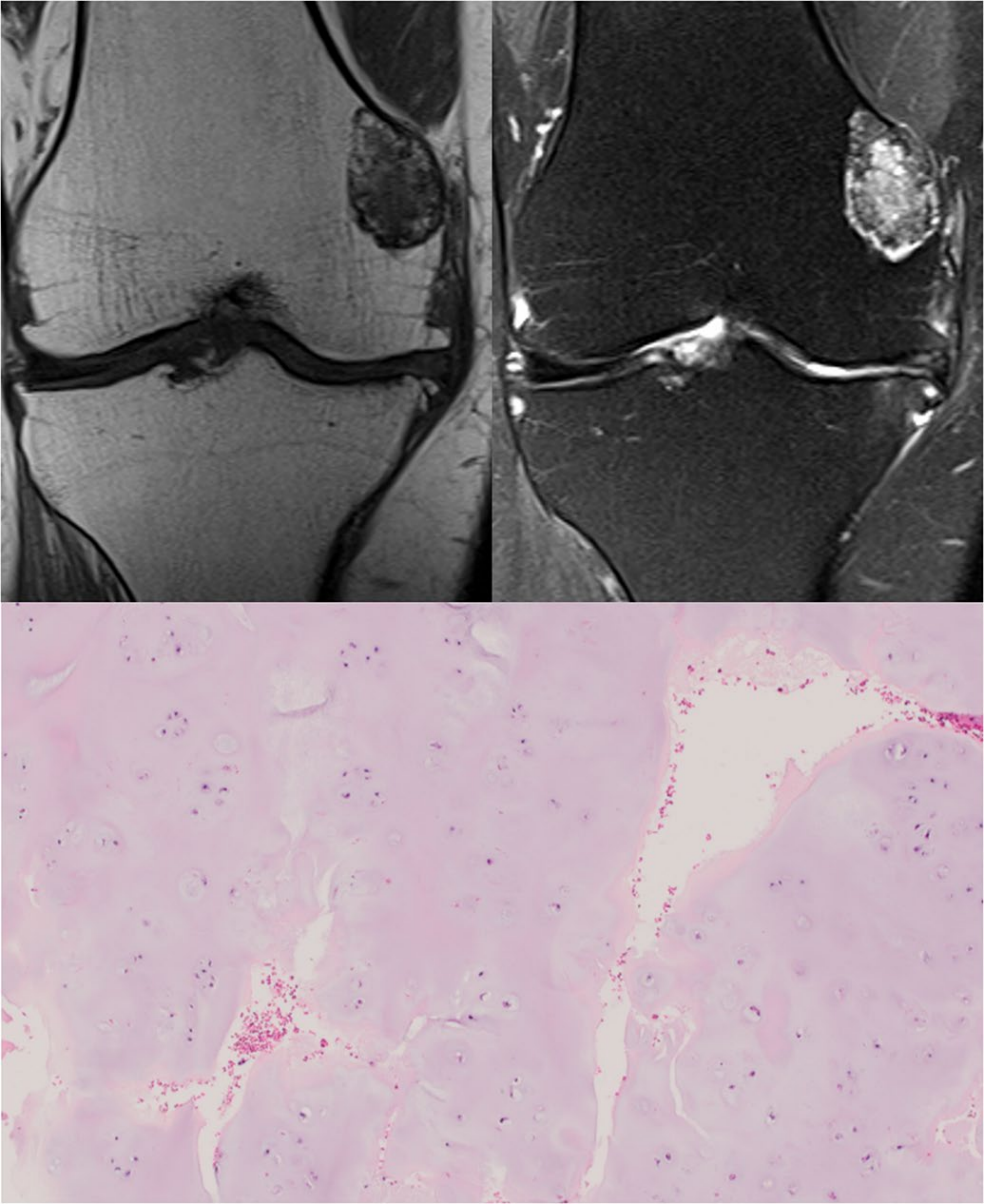
Sixty-five-year-old female with enchondroma. Coronal T1 (top left) and PDFS (top right) images show a well-defined eccentrically positioned metaepiphyseal lesion demonstrating chondral signal characteristics with interspersed fat signal. Associated endosteal scalloping which can be present in eccentric lesions. No cortical breach or extraosseous extension. Paucicellular lobules of hyaline cartilage with bland chondrocytes (bottom) (4 × magnification. H&E stain)

Most medullary cartilaginous tumours are diametaphyseal, but enchondromas occasionally occur in the epiphysis, and ACTs sometimes extend towards the end of the bone [59]. These low-grade tumours appear lobular, with variable chondral (ring and arc) mineralisation. MRI shows features of hyaline cartilage: high signal on fluid-sensitive sequences and low signal on T1WI; punctate low signal due to matrix mineralisation; peripheral and septal enhancement [19] (Fig. 10). Foci of entrapped fatty marrow suggest a low-grade lesion [60] (Fig. 10).

**Fig. 11:**
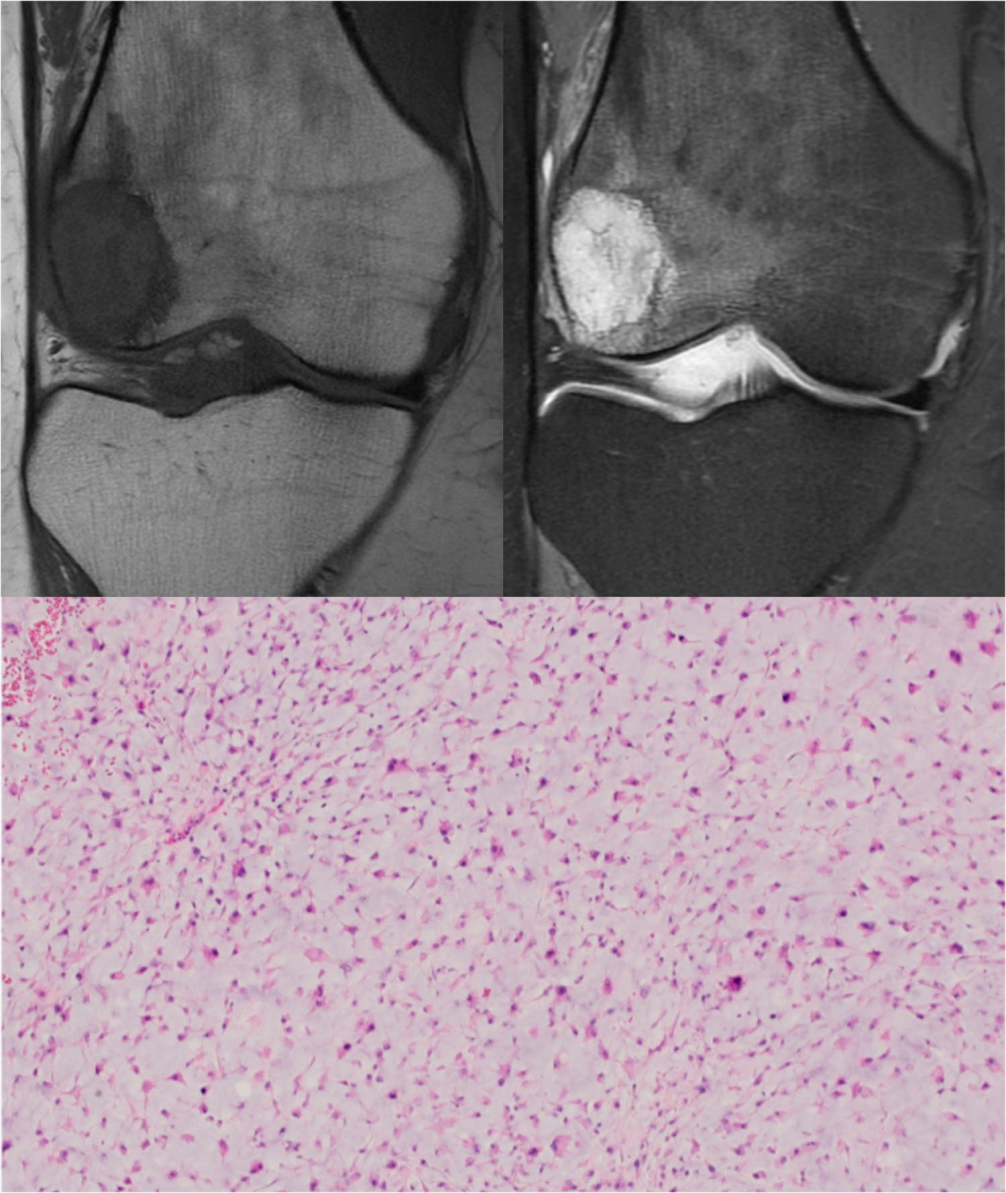
Forty-four-year-old female with central chondrosarcoma Grade 2. Coronal T1 (top left) and PDFS (top right) images show a subarticular lesion demonstrating chondral signal characteristics. The associated perilesional oedema raises concerns for a high-grade tumour. Severely atypical chondrocytes with high cellularity, set within a myxoid matrix (bottom) (4 × magnification, H&E stain)

Distinction of En and ACT is aided by lesion length (> 5 cm) (a commonly used but debated threshold) and deeper endosteal scalloping (both favouring ACT), but epiphyseal enchondromas located eccentrically can cause deep scalloping [59]. Intramedullary cartilaginous tumours in the epiphysis are rare but should be treated with suspicion: in one study, 16% of 95 CS were epiphyseal compared to only 3% of 92 enchondromas [61] (Fig. 11; Fig. S2).

Central CS is common in the distal femur, often affecting the knee [62]. CS can develop from an enchondroma (secondary central CS), the risk of which is greater in patients with enchondromatosis (most commonly Ollier disease and Maffucci syndrome) [63]. Radiographic features that suggest high-grade CS include an elongated tumour with deep endosteal scalloping, cortical thickening or destruction, bone expansion, periostitis, marrow oedema and an extraosseous mass [64]. Typical chondral features (matrix mineralisation and septal/nodular enhancement) are often partially retained.

**Fig. 12:**
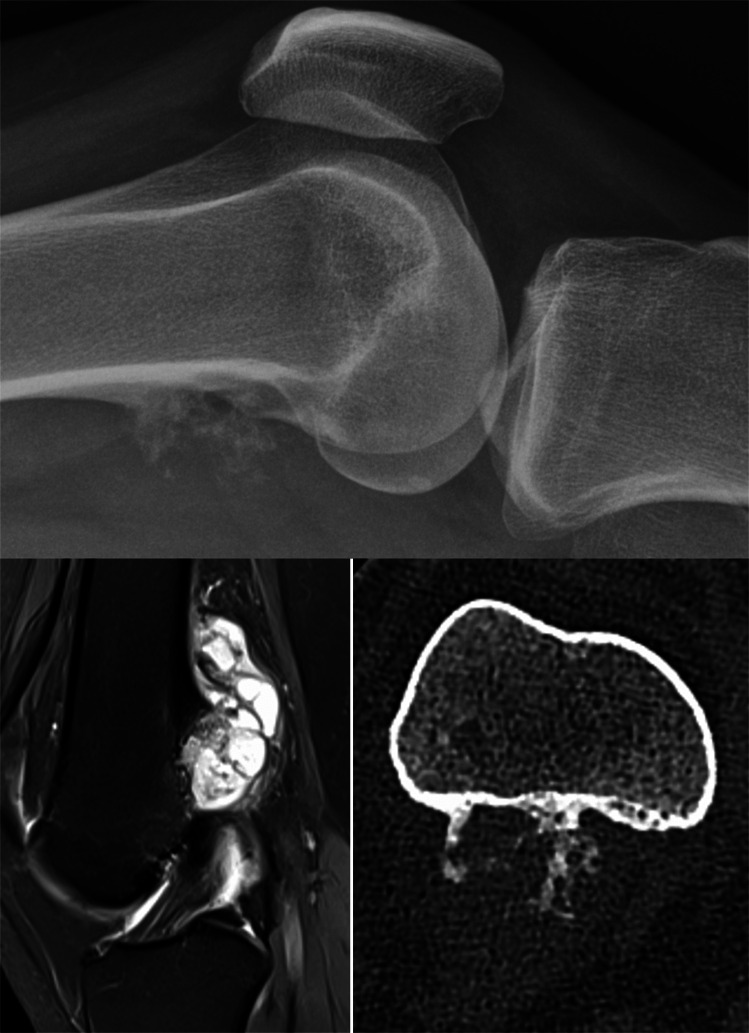
Twenty-four-year-old female with periosteal chondrosarcoma. Lateral radiograph (top) demonstrates a mineralised surface lesion with subtle cortical erosion and an associated cortical buttress. Sagittal PDFS image (bottom left) reveals a lobular subperiosteal surface lesion with chondral signal characteristics extending beyond the margins of the superior cortical buttress. Erosion of the underlying cortex is present without significant intramedullary extension. Axial CT image (bottom right) shows a low attenuation surface lesion with regions of intralesional matrix mineralisation and incomplete peripheral mineralisation. Adjacent sclerosis and deep cortical erosion that can be associated with chondrosarcoma are not seen

Cartilaginous tumours also arise deep to the periosteum (periosteal chondromas (Fig. S3) and CS). In common with other periosteal lesions, they cause pressure erosion/scalloping with a surrounding cortical buttress, occasionally partially covered by a shell of bone (Fig. 12). Features suggesting CS include size > 5 cm and cortical invasion, but marrow involvement is rare [46, 65].

Enchondromas and ACTs in the extremities are usually managed conservatively with clinical and imaging surveillance. Symptomatic or growing lesions can be treated with intralesional curettage with or without bone graft or local adjuvants. High-grade CS requires wide surgical excision with negative margins. Cartilaginous tumours of the flat bones are generally considered to be more aggressive, and even low-grade chondrosarcomas are therefore treated with resection [66]. Radiotherapy and chemotherapy have limited roles in the initial (curative) treatment of conventional chondrosarcomas.

### Osteochondroma (OC)/hereditary multiple exostoses (HME)

**Fig. 13:**
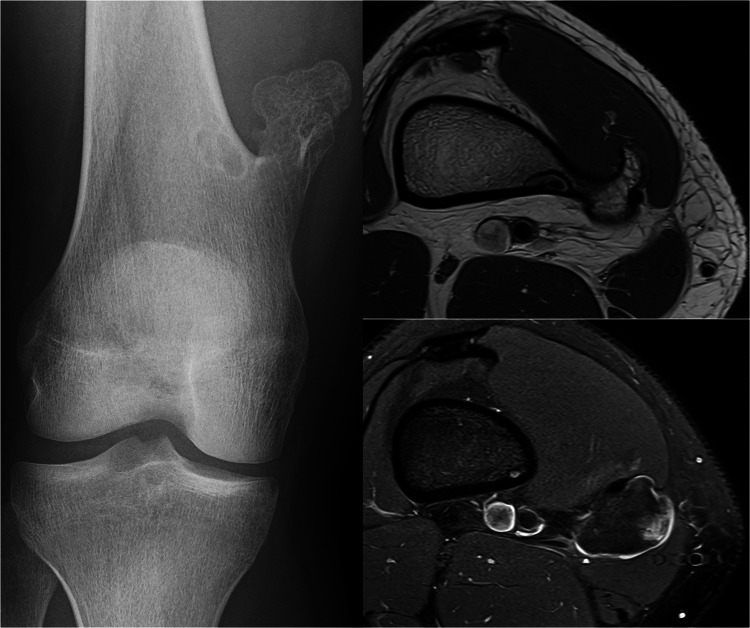
Eighteen-year-old male with pedunculated osteochondroma and non-ossifying fibroma. AP radiograph of the knee (left) shows a pedunculated osteochondroma arising from the medial distal femoral metaphysis, projecting away from the joint and demonstrating corticomedullary continuity. An eccentrically positioned NOF with central lucency and peripheral sclerosis is also present. Axial PD weighted image (top right) shows the pedunculated osteochondroma with corticomedullary continuity and moulding around the distal adductor magnus tendon. The NOF is seen in the posteromedial femoral cortex. Axial PDFS image (bottom right) confirms a slender, hyperintense chondral cap

**Fig. 14:**
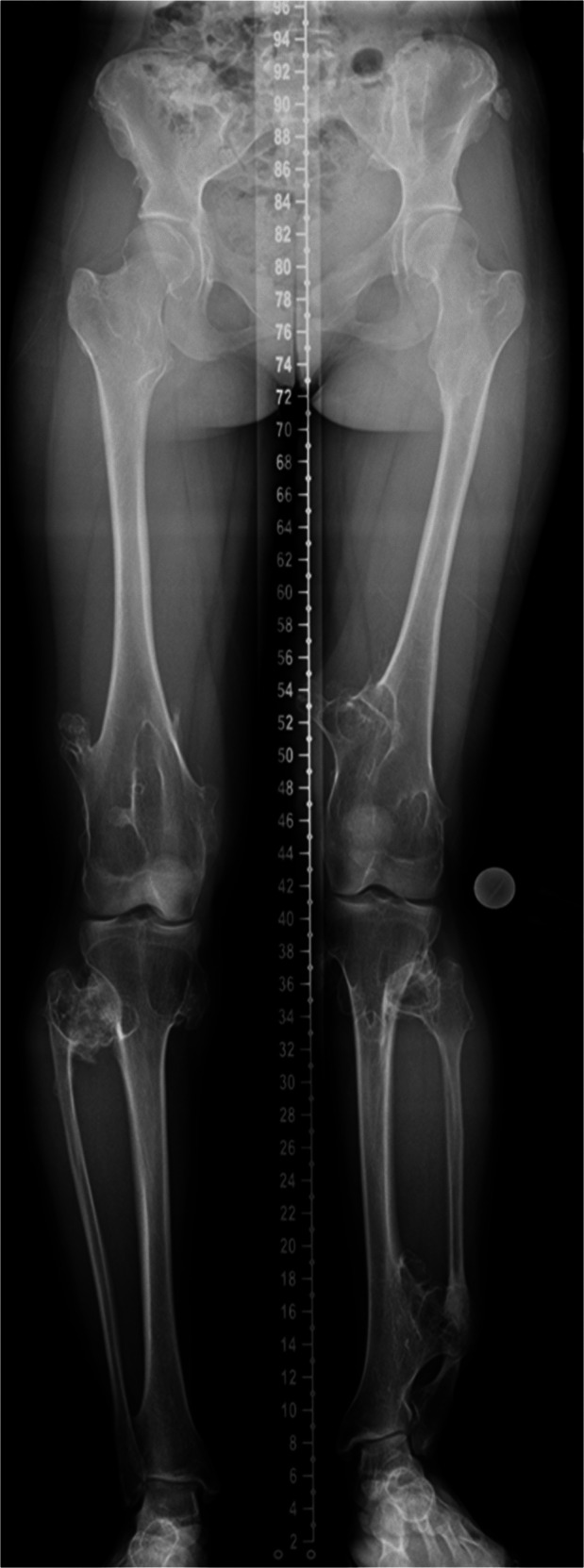
Twenty-one-year-old female with multiple hereditary exostoses. Long leg radiograph (with 3-cm block under the left leg) reveals multiple sessile and pedunculated osteochondromata, with evidence of osseous remodelling including metaphyseal widening, osseous bowing and growth disturbance with leg length discrepancy

OC are peripheral cartilaginous tumours consisting of a bony projection with an overlying chondral cap. They are thought to account for approximately 35% of benign bone tumours and are frequent in the knee region [67]. Approximately 85% are solitary (Fig. 13), the remainder occurring as multiple lesions in the autosomal dominant syndrome, HME (Fig. 14). OCs are caused by an inactivating mutation in the *EXT1* and *EXT2* genes [68].

**Fig. 15:**
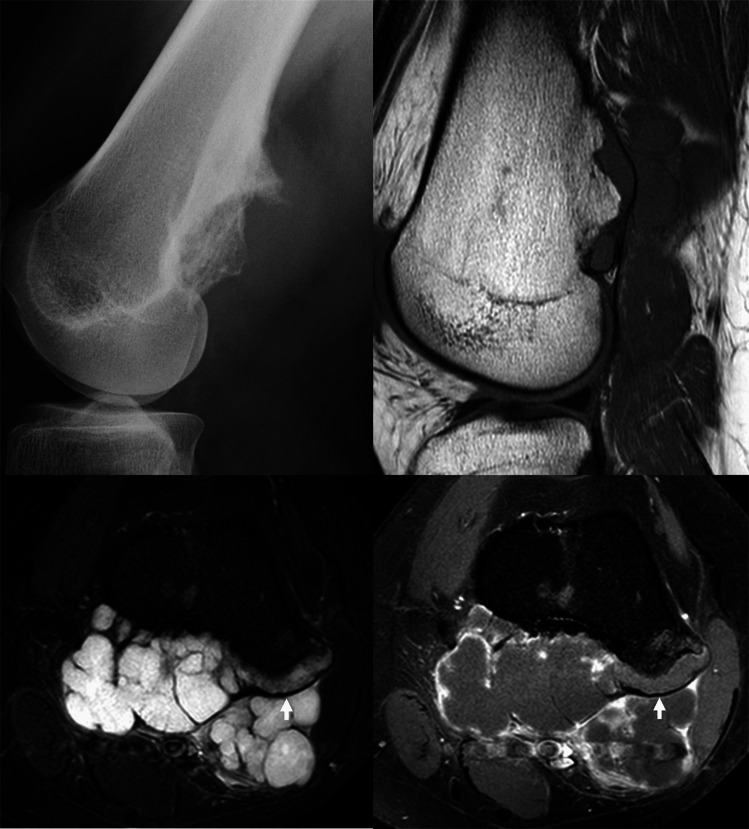
Twenty-six-year-old female with peripheral atypical cartilaginous tumour. Lateral radiograph (top left) demonstrates a surface lesion with regions of mature ossification, lucency, cortical erosion and prominent underlying sclerosis. Sagittal T1-weighted image (top right) demonstrates mature ossification corresponding to the residual osteochondroma with a non-thickened low signal cartilage cap, in addition to an overlying lobular mass showing similar signal. Axial PD (bottom left) and T1FS post-contrast (bottom right) images reveal lobular chondral signal tissue with peripheral, septal and nodular post-contrast enhancement which extends medial and superficial to the residual osteochondroma and non-thickened chondral cap (arrow)

Imaging shows a bony mass demonstrating continuity with the cortex and medulla of the host bone, frequently seen on radiographs if the OC is attached by a stalk (pedunculated) (Fig. 13). CT or MRI may be needed to show corticomedullary continuity in lesions with a wider bony origin (sessile OC) [19]. The cartilage cap is hyperintense on fluid-sensitive MRI sequences and is usually only a few millimetres thick: a cartilage cap of > 2 cm in adults would raise the suspicion of malignant transformation into a peripheral surface ACT/chondrosarcoma (Fig. 15), with transformation rates higher in HME [68, 69]. MRI of symptomatic OC may also show impingement of muscles, tendons and nerves and sometimes an overlying bursa [70]. Vascular compression at the knee is rare but can lead to venous thrombosis and a popliteal pseudoaneurysm [71].

Asymptomatic osteochondromas require no treatment; excision is performed for pain, functional limitation, deformity or neurovascular compromise, removing the lesion at the cortex and ensuring removal of the cartilage cap and perichondrium. HME may require repeated surgical interventions for impingement and deformities, including osteotomies or guided growth techniques [67, 72]. In children, careful timing of surgery is important to avoid growth plate injury [67, 72].

### Aneurysmal bone cyst (ABC)

Whilst originally considered a non-neoplastic lesion, primary ABCs were shown to be neoplasms following the identification of *USP6* genetic alterations [73].

**Fig. 16:**
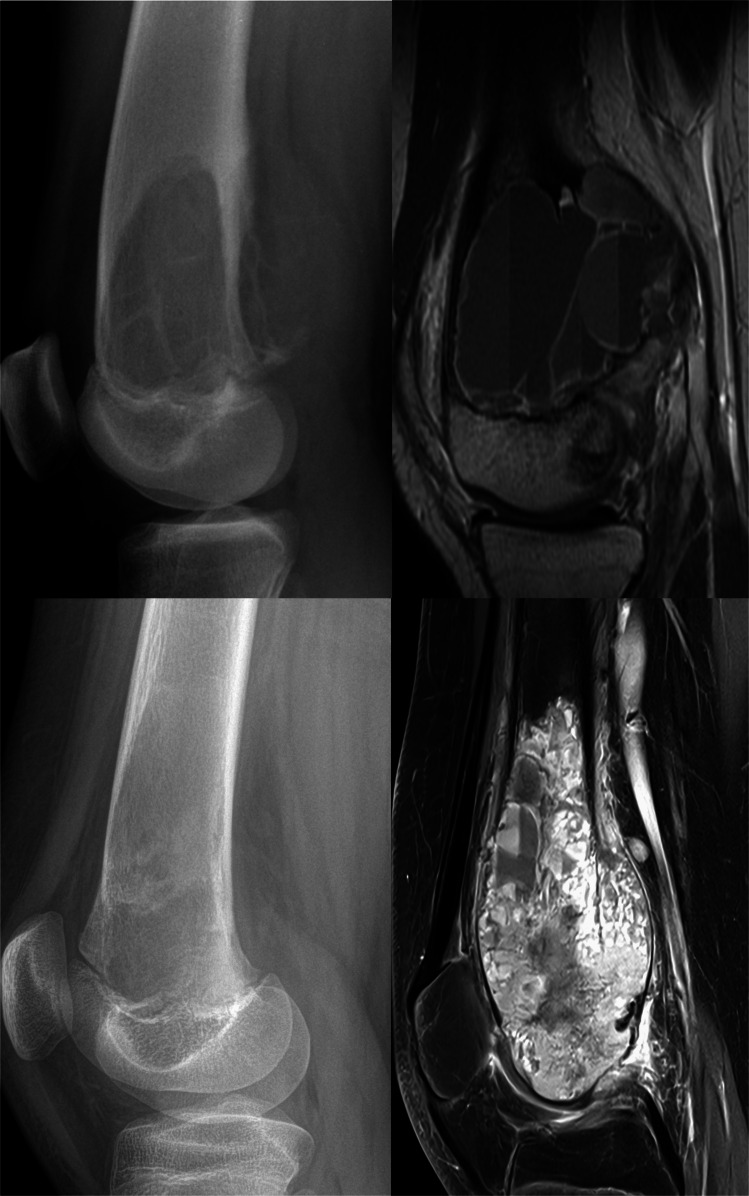
Comparison between ABC and telangiectatic osteosarcoma. Fourteen-year-old female with aneurysmal bone cyst (top images). Lateral radiograph (top left) of the knee demonstrates a well-defined expansile lytic lesion within the distal femoral metaphysis with a significant posterior component resulting in an attenuated cortex which is imperceptible posteriorly. Sagittal T1-weighted image post-contrast (top right) reveals a multiloculated lesion comprising entirely of multiple fluid–fluid levels with peripheral and septal enhancement, and importantly, no significant enhancing soft tissue component. Twelve-year-old female with telangiectatic osteosarcoma (bottom images). Lateral radiograph (bottom left) demonstrates an ill-defined distal femoral metadiaphyseal lytic lesion with regions of faint lesional sclerosis. Sagittal T1FS post-contrast image (bottom right) shows a complex tumour with multiloculated components, fluid–fluid levels in addition to prominent areas of nodular enhancing solid lesional tissue

Benign, malignant or non-neoplastic conditions can show ABC-like changes. ABCs contain multiple blood-filled spaces, giving rise to characteristic fluid–fluid levels on MRI [74] (Fig. 16). ABCs can be diagnosed in a wide age range but are more frequent in the first two decades of life, accounting for 1–2% of primary bone tumours and showing a slight female predominance [75].

In the appendicular skeleton, ABCs typically present as an intramedullary lesion in the metaphysis of a long bone, with the distal femur and proximal tibia being common locations [76]. Surface lesions, either intracortical or subperiosteal, are usually diaphyseal [76].

They show initial osteolysis and periosteal elevation, progressing to aneurysmal expansion of the bone such that an external border may not be radiographically visible. CT may show a thin cortical shell and MRI a low signal margin [77]. Subsequent stabilisation results in better definition, thicker peripheral ossification and internal septation [75].

Thin septal enhancement is typical in ABCs (Fig. 16); a significant solid component suggests another primary lesion with ABC-like change [78]. An important lesion which should be differentiated from ABC is telangiectatic osteosarcoma [79] (Fig. 16).

Treatment of primary ABC includes percutaneous sclerotherapy, selective arterial embolisation, curettage (with adjuvants and grafting) and denosumab, depending largely on size and location [80]. Systemic therapy with denosumab should be reserved for children with unresectable lesions.

In addition, a range of benign (Fig. S4) and malignant (Fig. S5) tumours with no distinct anatomical preference may show knee involvement, and imaging frequently offers clues to the diagnosis.

## Non-neoplastic lesions

### Subarticular

#### Cystic lesions

*Subarticular cysts/geodes* affect up to 30% of patients with osteoarthritis [81]. The aetiology may involve subchondral bone injury by repeated passage of joint fluid through a defective articular surface or infarction of unprotected marrow [81, 82]. The cysts typically lack a synovial lining. Reactive fibrous, cartilaginous or bony proliferation may obscure the defect on imaging [83].

*Intraosseous (IO) ganglia* do not communicate with the adjacent joint space, nor do they contain synovial fluid and are not associated with osteoarthritis [82, 84]. They are commonly found at ligament attachments secondary to mucoid degeneration at the bone/ligament interface or intraosseous extension of an adjacent soft tissue ganglion [82].

**Fig. 17:**
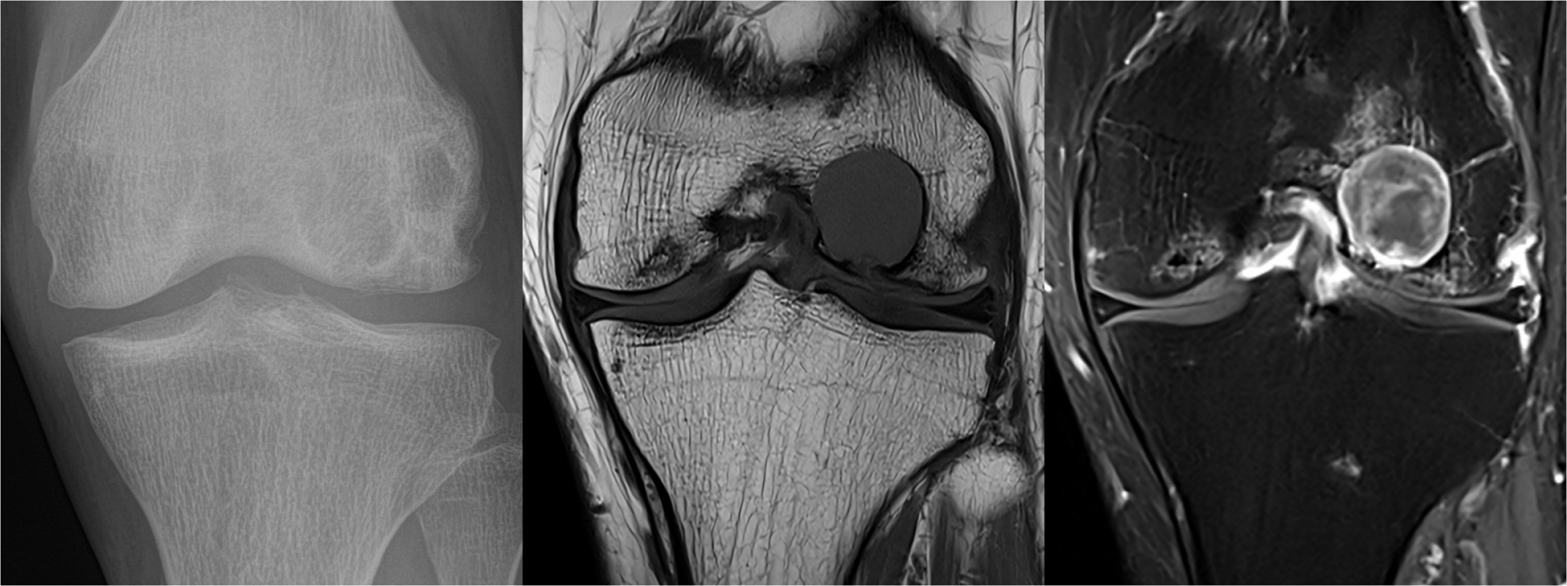
Twenty-five-year-old male with subchondral cyst. AP radiograph of the knee (left) with lateral tibiofemoral compartment osteoarthritis and subarticular cystic change underlying the lateral femoral condyle subchondral bone plate. Coronal T1-weighted image (centre) shows well-defined cystic signal characteristics underlying focal distortion of the subchondral bone plate. Incidental note is made of osteonecrosis involving the medial femoral condyle which demonstrates central fat signal. Coronal T1FS post-contrast image (right) highlighting predominantly thin peripheral enhancement

Cystic lesions are usually identified during imaging evaluation of knee pain. A large geode may become symptomatic following pathological fracture or haemorrhage [84]. They can mimic subarticular tumours. Radiographically, subchondral cysts are lucent with thin sclerotic margins, typically at weight-bearing articular surfaces, showing no significant bone expansion (Fig. 17). A periosteal response may be observed following fracture. IO ganglia have similar appearances but localise to ligament attachments.

These lesions usually demonstrate cystic MR signal characteristics, but hyperintensity on T1WI may be seen due to haemorrhagic or proteinaceous content. There may be adjacent bone marrow oedema. Fibrous content, septations, intervening bony trabeculae and synovial thickening may alter the appearance of these cysts [84]. Fluid–fluid levels and periostitis may be visible when complicated by fracture [84]. Thin peripheral enhancement following contrast administration is commonly seen [85]. (Fig. 17).

*Sub-periosteal ganglia* are caused by mucoid degeneration of the periosteum [86]. They often occur at the proximal tibia near the pes anserinus and are more common in males in the forth to fifth decades [87]. On MRI, they appear as well-defined cystic lesions in the superficial cortex, with cortical scalloping but no medullary involvement (Fig. S6). They may mimic periosteal chondral tumours, periosteal ABCs or haematomas [86, 87].

#### Trevor disease (TD)

TD (dysplasia epiphysealis hemimelica, DHE) is a rare developmental disorder characterised by asymmetric epiphyseal osteocartilaginous proliferation. It is more common in males and can affect multiple joints on the same side of the body, usually in the lower limbs, particularly the knee and ankle [88, 89].

**Fig. 18:**
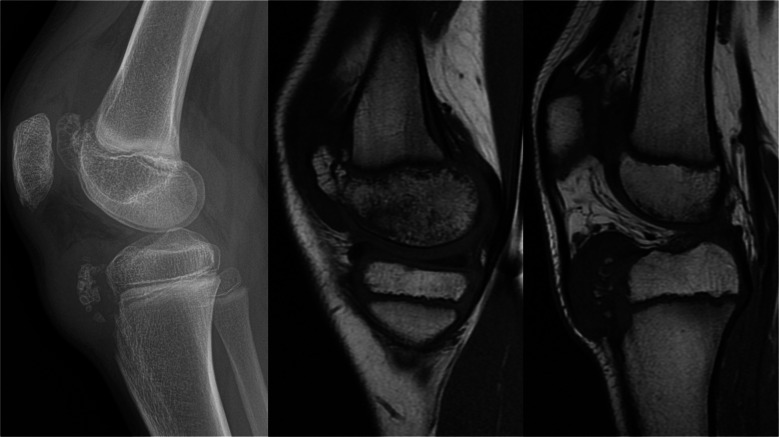
Eight-year-old female with Trevor disease. Lateral knee radiograph (right) demonstrates an ossified mass adjacent to the distal femoral epiphysis with distortion of the quadriceps insertion. Multiple discrete areas of ossification in the region of the tibial apophysis with distortion of the patellar tendon contour. T1-weighted sagittal images (centre and left) confirm marrow fat signal within the regions of ossification within surrounding low T1 un-mineralised cartilaginous components

Radiographically, there is asymmetric epiphysial overgrowth and multiple osteocartilaginous masses: MRI shows hyperintense cartilage signal on T2WI. When ossified, lesions can show corticomedullary continuity mimicking osteochondromas and may fracture [88, 89] (Fig. 18).

#### Avascular necrosis (AVN)

AVN (osteonecrosis) reflects marrow infarction and is commonly seen in subarticular locations in the distal femur and proximal tibia [90]. There are numerous causes, including trauma, steroid administration and alcohol use disorder, but it is frequently idiopathic [91].

Early AVN shows patchy sclerosis with subtle osteopenia of the surrounding vascularised bone. Later, a serpentine sclerotic border forms around the infarct, representing a reactive interface and new bone formation (the ‘zone of creeping substitution’) [91].

**Fig. 19:**
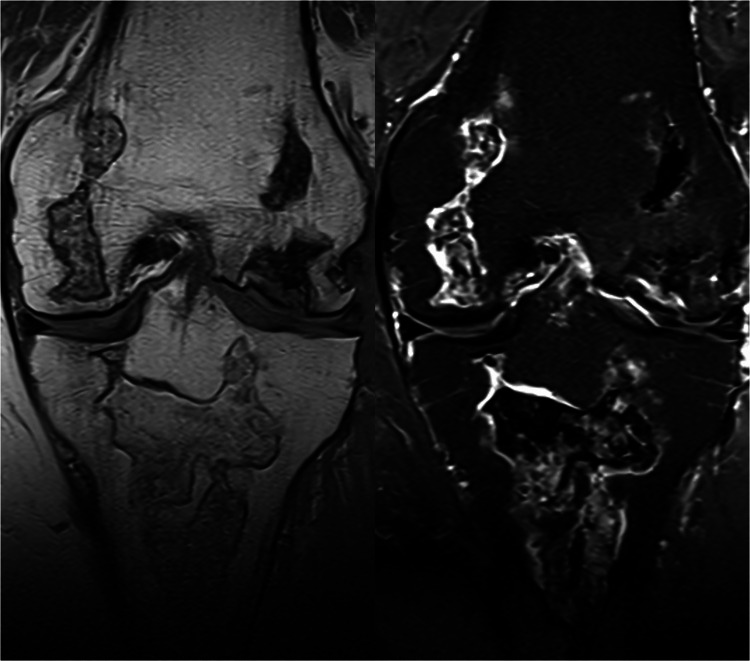
Sixty-year-old male with multifocal osteonecrosis. Coronal T1 (left) and STIR (right) images demonstrate geographic areas of altered signal within the distal femur and proximal tibia. These areas demonstrate central fat signal or rarely heterogeneous low signal and are outlined by peripheral low signal and adjacent increased STIR signal suggestive of granulation tissue (a ‘double line’ sign). Associated lateral femoral condyle subchondral collapse with depression of the subchondral bone plate and underlying fluid-signal fracture cleft

MRI can detect changes as early as 1 week after ischaemic insult, demonstrating an irregular, demarcated focus of fatty marrow [91]. The margin consists of peripheral low signal on T2WI (due to sclerosis) and inner hyperintense signal (which corresponds to a vascularised reactive zone), forming the ‘double line’ sign [92] (Fig. 19). Internal haemorrhage and cystic degeneration may add complexity [91].

Subarticular AVN can lead to fracture and articular collapse, which may cause secondary osteoarthritis [91].

Malignant transformation of AVN is extremely rare [93] (Fig. S5). It most commonly occurs in the diametaphysis of the distal femur rather than in subarticular regions [94]. Aggressive bone lysis and frequently an extraosseous mass destroy an area of AVN [93]. Pleomorphic sarcoma and osteosarcoma are the most frequently reported malignancies [93].

#### Subchondral insufficiency fractures (SIF)

Fractures of the subchondral bone are most frequent at the weight-bearing surface of the medial femoral condyle and are associated with meniscal tears, osteoarthritis and osteoporosis [95–97]. Most cases resolve spontaneously, but some are associated with subchondral collapse, usually if there is adjacent osteonecrosis [96].

Patients typically present with spontaneous pain with no clear history of trauma. Early SIFs are often radiographically occult, but there may be subtle subchondral lucency [96, 97]. SIFs associated with osteonecrosis may show subchondral sclerosis and articular surface depression [97].

**Fig. 20:**
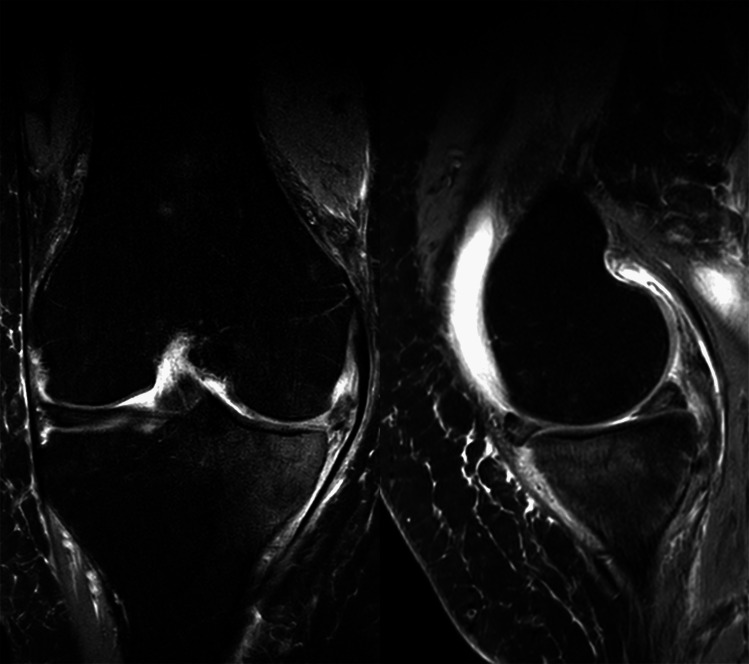
Eight-seven-year-old female patient with subchondral insufficiency fracture. Coronal (left) and sagittal PDFS (right) images demonstrate focal medial tibial plateau subarticular oedema-like marrow signal underlying subtle thickening/depression of the subchondral bone plate. Subtle underlying low signal fracture line with degenerative tearing and extrusion of the medial meniscal body

MRI typically reveals bone marrow oedema-like signal in the subchondral bone and oedema-like hyperintensity in soft tissue (Fig. 20), particularly overlying the metaphysis (the metaphyseal burst sign) [98].

A thin, irregular, hypointense subchondral fracture line may be observed (Fig. 20). Some cases demonstrate a subchondral hypointense band on T2WI. In reversible SIF, this band typically measures less than 2 mm in thickness; in irreversible SIF, the band is thicker than 4 mm and longer than 14 mm, indicating a high risk of osteonecrosis [96, 97]. A fluid-signal cleft suggests an unstable osteochondral fragment, which may progress to articular collapse [97].

### Metaphyseal

#### Distal femoral cortical irregularities (DFCI)

These self-limiting fibro-osseous lesions are common incidental findings, also called cortical desmoids/tug lesions and Bufkin lesions. They most commonly affect individuals aged 10–15 years [99], are typically located on the posteromedial aspect of the supracondylar distal femur and are bilateral in up to 35% of cases.

They are thought to result from excessive cortical remodelling due to repetitive traction at the gastrocnemius or adductor magnus attachments. Their higher prevalence in athletes, larger size in the dominant limb, increased frequency after rapid growth spurts and histological findings of osteoclastic activity and cortical resorption support this hypothesis [100]. They may occasionally cause activity-related pain [99, 100].

**Fig. 21:**
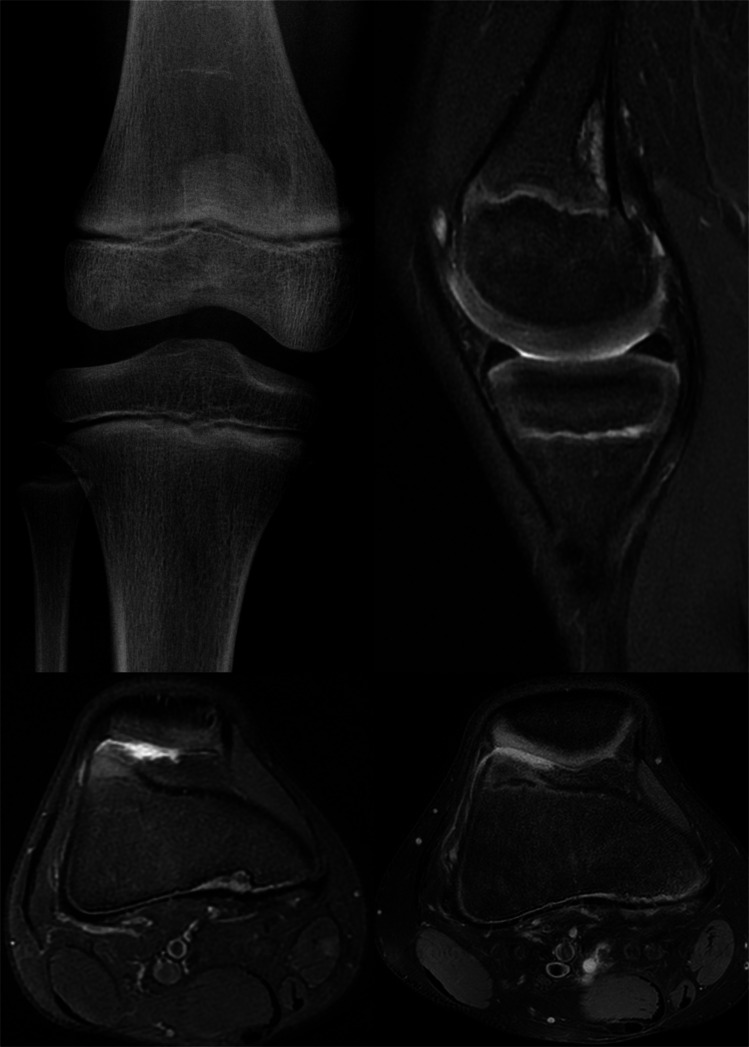
Eight-year-old male with distal femoral cortical irregularity (DCFI). AP knee radiograph (top left) demonstrates a well-defined eccentrically located lucency within the medial distal femoral metaphysis with a peripheral sclerotic margin. Sagittal and axial PDFS (top right and bottom left) images reveal a corresponding area of signal change with a peripheral low signal margin, underlying the medial head of gastrocnemius enthesis. Interval axial PDFS image 4 years later (bottom right) demonstrate resolution of the DFCI which is now difficult to demonstrate from the normal distal femoral metaphyseal stripe of vascular subperiosteal fibrous tissue

Radiographs show eccentric cortical irregularity and lucency (Fig. 21). On MRI, there is hyperintense cortical irregularity with a low-signal-intensity peripheral rim on T2WI, most commonly at the posteromedial distal femur, underlying the medial gastrocnemius origin (Fig. 21). Periostitis, mild bone marrow, soft tissue oedema and gastrocnemius hyperintensity are common [99]. DFCIs can mimic non-ossifying fibromas (rarely located at this site) and surface osteosarcomas [99].

### Traumatic lesions

#### Myositis ossificans

Myositis ossificans (MO) is often post-traumatic, but a history of injury is lacking in 50% [101]. It also occurs after spinal cord or brain injury and as part of fibrodysplasia ossificans progressiva [101]. Early MO appears aggressive due to the extensive soft tissue hyperintensity and poorly defined mass; mature MO occurring near bone can mimic parosteal osteosarcoma [101]. Greater peripheral density (the ‘zoning’ phenomenon) and lack of an intervening lucent plane suggest MO, but close imaging observation is mandatory, occasionally requiring biopsy until the diagnosis is established [101].

#### Sub-periosteal haematomas (SPH)

**Fig. 22:**
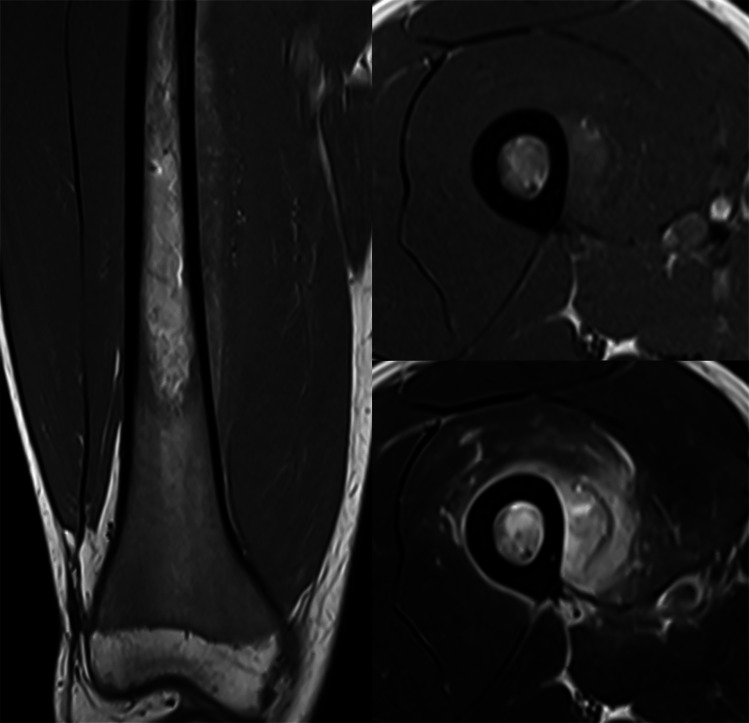
Twenty-one-year-old male with subperiosteal haematoma and history of sickle cell anaemia. Coronal T1 (right), axial T1 (top right) and axial T2 (bottom right) images show a subperiosteal lesion with T1 hyperintensity suggestive of blood products with associated periostitis and adjacent muscle oedema

SPH are most common in the calvarium and pelvis but can be seen in long bones [102]. In addition to trauma, haemophilia, vitamin C deficiency and liver disease may be contributory [102]. Elevation of the periosteum can be lenticular or circumferential, with the MR signal of intervening tissue reflecting blood products (Fig. 22). SPH eventually ossifies (Fig. S7).

Ultrasound demonstrates a heterogeneous avascular subperiosteal mass containing lace-like echoes. MRI shows a heterogeneous fluid collection or mass deep to the stripped hypointense periosteum. Gradient echo images may exhibit a blooming artifact [102]. An important differential consideration is periosteal neoplasms, especially periosteal osteosarcoma.

#### Brodie abscess (BA)

Subacute osteomyelitis may form an intraosseous (Brodie) abscess. It is usually located in the metaphyses of long bones, related to the physis [103]. It can manifest at any age, most commonly in children aged 2–12 years [103].

**Fig. 23:**
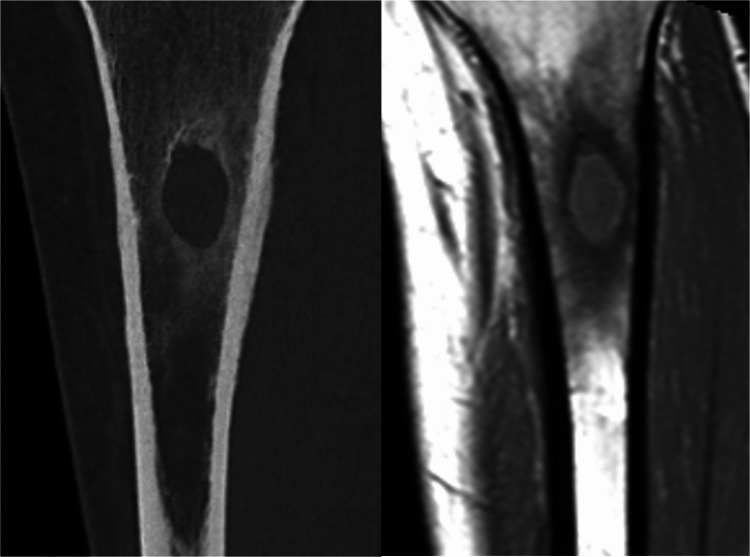
Thirty-eight-year-old male with Brodie’s abscess. Coronal CT reconstruction (left) shows a well-defined lytic lesion within the proximal tibial metaphysis with a peripheral sclerotic margin, adjacent sclerosis and periosteal new bone formation medially. Corresponding coronal T1-weighted MRI (right) shows a relatively well-defined lesion with central hypointense signal, peripheral hyperintense margin (‘penumbra’ sign indicating granulation tissue) and surrounding oedema-like signal change

Radiographically, there is a lytic lesion with a thin sclerotic margin, occasional periosteal reaction and curvilinear lucent extension towards the growth plate that may be visible [104]. On T1WI, BA shows central hypointensity with a hyperintense peripheral rim due to vascularised granulation tissue (‘penumbra sign’) and surrounding marrow oedema [105] (Fig. 23). The peripheral rim demonstrates intense enhancement. A penumbra sign is helpful in differentiating BA from tumours [105].

## Miscellaneous

### Paget disease (PD)

PD of bone is caused by both increased bone resorption and formation due to aberrant osteoclast function [106]. It affects an aging population, typically of Northern European descent, and appears to be reducing in prevalence [107]. The femur is affected in 25%–55% of cases and may fracture [106, 108].

**Fig. 24:**
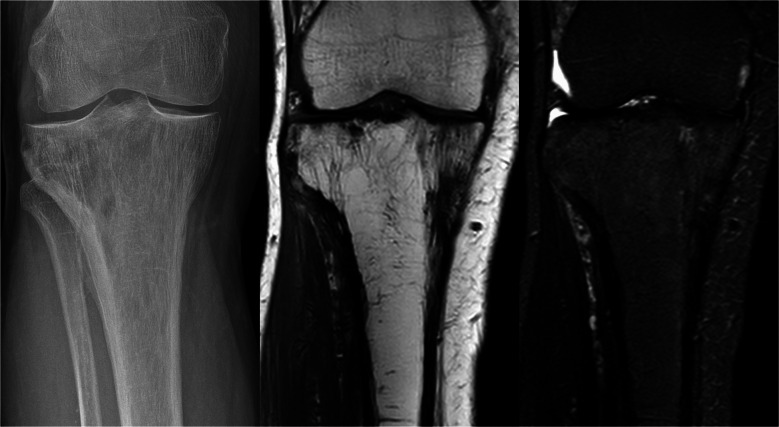
Eighty-four-year-old male with Paget disease of the tibia. AP knee radiograph (left) shows trabecular coarsening, cortical thickening and expansion of the lateral tibial plateau. Coronal T1 (centre) and STIR (right) images confirm the absence of an underlying lesion and again illustrate the presence of trabecular coarsening, cortical thickening and expansion of the lateral tibial plateau

The initial lytic phase reflects excessive osteoclastic activity and almost invariably begins in the subchondral bone of the epiphysis, extending into the metadiaphysis with a flame-shaped leading edge (‘blade of grass’). Subsequently, a mixed phase results in sclerosis, with coarsening and thickening of bony trabeculae along the lines of stress. In the final blastic or late inactive phase, osteoblastic activity declines and there is often bone expansion [109, 110]. MRI shows at least partial preservation of fatty marrow, which is diagnostically useful (Fig. 24), but in the early phases, marrow and cortical signal can be heterogeneous, mimicking a tumour.

### Brown tumour of hyperparathyroidism

**Fig. 25:**
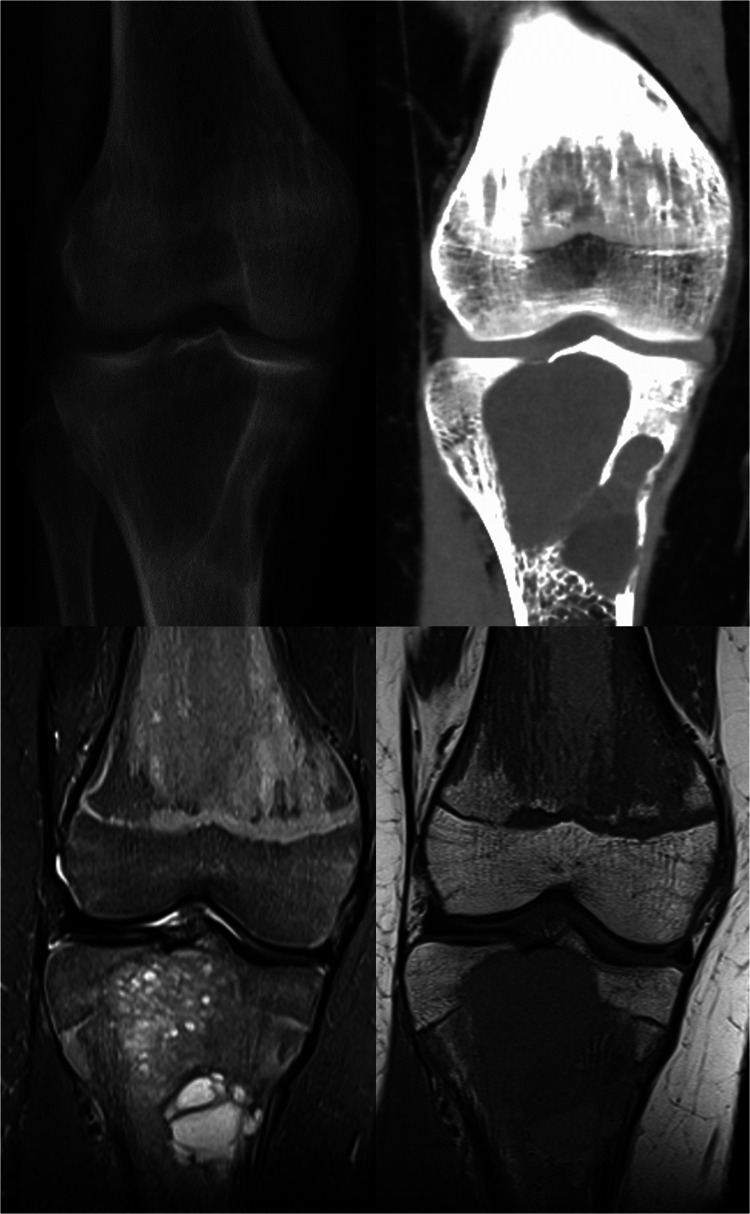
Sixteen-year-old female with Brown tumour. Coronal average intensity projection radiograph-like (top left) and CT reconstruction (top right) images showing lytic lesions within the proximal tibia of varying attenuation. Expansion related to the inferior lesion results in endosteal scalloping and deficiency of the medial cortex. Coronal STIR (bottom left) and T1-weighted (bottom right) images show complex corresponding signal characteristics with sub-physeal resorption best appreciated related to the distal femoral physis

Excessive osteoclastic bone resorption in hyperparathyroidism causes lytic foci [107]. Due to earlier diagnosis, this is now rare in developed countries [111]. Brown tumours are slightly more common in primary than in secondary or tertiary hyperparathyroidism and are more commonly seen in flat bones than around the knee joint [107, 112]. Single or multiple expansile lytic lesions with or without sclerotic margins are seen on radiographs [107, 113]. On MRI, they may appear solid, cystic or complex with internal haemorrhage and fluid–fluid levels consistent with ABC-like change (Fig. 25). There are usually features of an underlying metabolic bone disease [107, 112].

### Melorheostosis

**Fig. 26:**
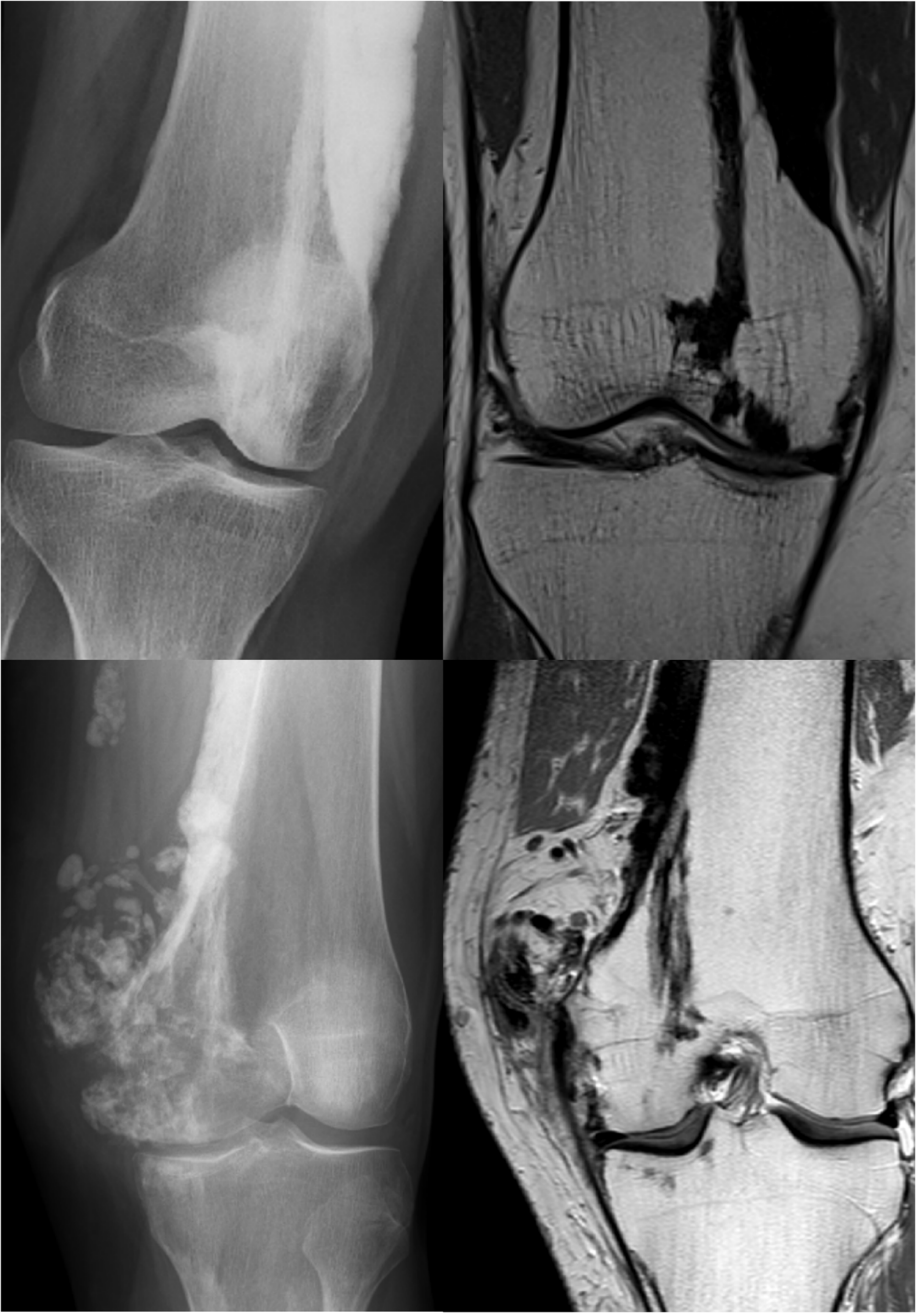
Forty-seven-year-old female with melorheostosis (top images). AP knee radiograph (top left) and coronal T1-weighted image (top right) reveal cortical and medullary hyperostosis with the medullary component appearing linear and striated, and more prominent in the epiphysis. Fifty-four-year-old male with melorheostosis (bottom images). AP knee radiograph (bottom left) and coronal T1-weighted image (bottom right) demonstrate a similar appearance, in addition to para-articular soft tissue involvement which (as in this case) is typically not attached to the underlying bone and can also contain fat or fibrovascular tissue. The appearances in both cases are consistent with a mixed sclerosing dysplasia, with additional features of osteopathia striata

Melorheostosis is a non-hereditary sclerosing dysplasia, frequently presenting in young patients and commonly in the lower extremities [114]. The classic ‘dripping candle wax’ appearance (cortical hyperostosis) typically extends from proximal to distal and can cross joints. Endosteal involvement may occur in later stages of the disease. It can mimic an osteoma, co-exist with other sclerosing dysplasias and extend to soft tissue [114, 115]. A surface bone lesion of cortical density should be differentiated from parosteal osteosarcoma, which is usually possible morphologically. MRI shows low-signal-intensity hyperostosis and heterogeneous signal in soft tissue lesions (Fig. 26). Both CT and MRI are useful for evaluating mass effect or impingement caused by the soft tissue component [116].

### Focal periphyseal oedema zone (FOPE)

**Fig. 27:**
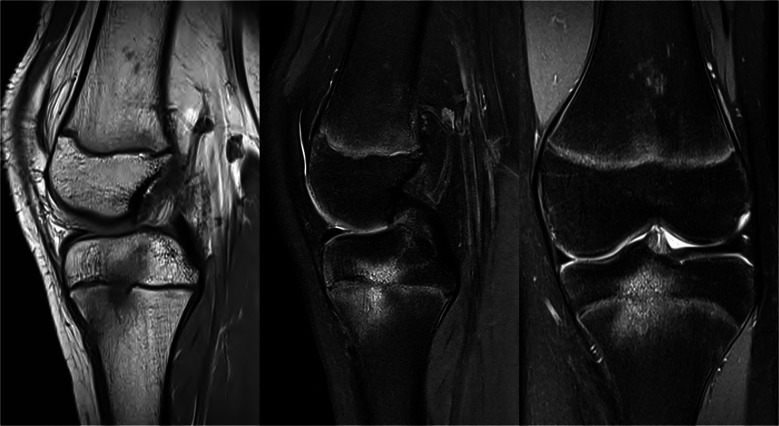
Thirteen-year-old female with FOPE zone. Sagittal T1 (left), PDFS (centre) and coronal PDFS (right) images reveal a focal region of oedema-like marrow signal centred on the physis which extends into the adjacent epiphysis and metaphysis. No abnormal physeal widening

Focal periphyseal oedema likely relates to the early stages of physeal fusion [117]. Oedema-like signal measuring between 2 and 27 mm is seen in the central physes of the knee, including the proximal fibula [117] (Fig. 27). It may be associated with pain, but should not be mistaken for tumour or infection [118].

## Reporting recommendations—‘dos and don’ts’

- The age of the patient and lesion location are valuable guides to diagnosis of tumours. Try to determine if the lesion is subarticular or metaphyseal, extending to the end of the bone.
- Remember that there are exceptions to the ‘rules’, for example metaphyseal GCT in young patients, chondroblastomas affecting a wide age range and diametaphyseal sites.
- Do look for joint involvement, intra-articular fractures and proximity to an articular surface, as this will guide management.
- Do look for imaging features that can aid diagnosis, such as marrow oedema-like signal (in osteoid osteoma and chondroblastoma), chronic haemorrhage (in GCT), mineralisation (bone-forming and chondrogenic tumours) and fluid–fluid levels (in primary ABC and ABC-like lesions, which are frequent at the knee—GCT and chondroblastoma).
- Do remember that, when dealing with a malignant lesion, imaging must assess the intra- and extraosseous disease in the entire bone.

## Conclusion

Bone tumours and tumour mimics commonly affect the knee. It is useful to consider lesions arising in subarticular bone as a distinct group, separate from lesions outside the knee extending to the end of the bone. Management of benign and malignant tumours is complicated if there is joint involvement, a significant extraosseous mass or an intra-articular fracture. Common subarticular neoplasms include giant cell tumour of bone and chondroblastoma; diametaphyseal malignancies that frequently extend to the knee include conventional osteosarcoma and chondrosarcoma. Common subarticular non-neoplastic entities include cysts, osteonecrosis and subchondral fractures. An accurate diagnosis can be made using the characteristic imaging features and site of origin and is vital for guiding management.

## Appendix 

Table 1

**Table 1:**
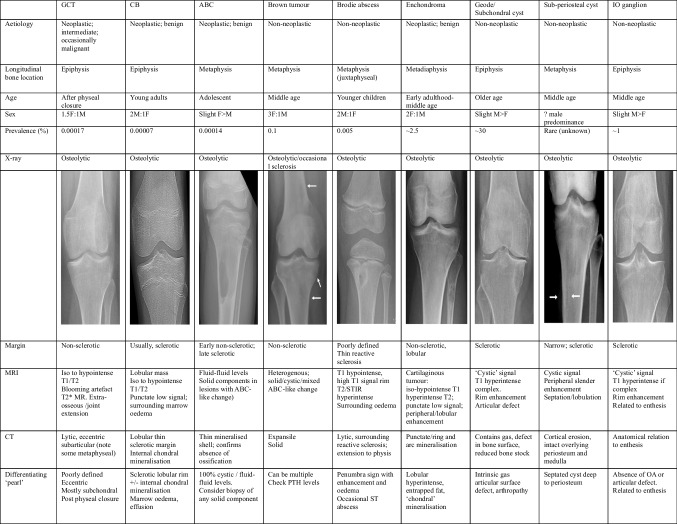
Osteolytic/lucent intra-osseous knee lesions

*MRI* magnetic resonance imaging, *CT* computed tomography, *GCT* giant cell tumour, *CB* chondroblastoma, *ABC* aneurysmal bone cyst, *IO* intra-osseous, *OA* osteoarthritis

## Supplementary Information

Below is the link to the electronic supplementary material.

Supplementary Material 1 55-year-old female with metastatic proximal fibular giant cell tumour (Same patient Fig. 3).  CT thorax lung (top) and mediastinal (bottom) windows of the same patient show a histologically proven left upper lobe GCT metastasis with associated Denosumab-induced ossification better appreciated on the mediastinal window (PNG 843 KB)

High Resolution Image (TIF 3.71 MB)

Supplementary Material 2 17-year-old female with central chondrosarcoma Grade 2. Sagittal T1 (top left), STIR (top right) and axial PDFS (bottom) images show a subarticular lesion demonstrating chondral signal characteristics with extraosseous extension into the intercondylar notch and prominent perilesional oedema, which together raise concerns for a high-grade tumour (857 KB)

High Resolution Image (TIF 3.79 MB)

Supplementary Material 3 17-year-old female with periosteal chondroma. Lateral radiograph (top) demonstrates a lucent surface lesion with pressure erosion/cortical scalloping and associated cortical buttress. Sagittal PDFS image (bottom left) reveals a well marginated lobular subperiosteal surface lesion with cortical buttressing and chondral signal characteristics.  Intramedullary extension makes differentiation form a chondrosarcoma difficult on imaging but walled off-marginal sclerosis is present. Axial CT (bottom right) confirms cortical erosion with an incomplete periosteal mineralised margin and intraosseous extension with an intramedullary lytic lesion showing peripheral marginal sclerosis (PNG 954 KB)

High Resolution Image (TIF 5.24 MB)

Supplementary Material 4 33-year-old male with intraosseous lipoma. AP radiograph (left) demonstrates a mildly expansile mixed lytic and sclerotic lesion centred within the medial femoral condyle with a peripheral sclerotic margin and internal calcification. Coronal T1 weighted image (right) reveals prominent regions of intralesional fat signal and low signal cystic foci suggestive of fat necrosis (PNG 1.25 MB)

High Resolution Image (TIF 7.02 MB)

Supplementary Material 5 Bone sarcoma. 56-year-old male with infarct associated high grade spindle cell sarcoma (left). Coronal T1 weighted image (left) demonstrates proximal tibial marrow infiltration with extraosseous extension on a background of multifocal osteonecrosis. 50-year-old male with high grade leiomyosarcoma (centre). AP knee radiograph (top centre) shows ill-defined lytic lesion centred on the medial proximal tibial metaphysis, with the superior component demonstrating a well-defined sclerotic margin. Extramedullary mineralisation with cortical erosion suggestive of a subperiosteal component.  Prominence of the tibial tuberosity reflects remodelling in keeping with previous Osgood-Schlatter Disease. Coronal T1 weighted image (bottom centre) shows marrow infiltration with the lesion demonstrating regions of differing signal characteristics.  Medial subperiosteal component with attenuation of the adjacent cortex. 62-year-old male with radiation induced osteosarcoma.  History of previous surgery and radiotherapy for sarcoma 40 years ago with subsequent intramedullary nail for insufficiency fracture (right). AP knee radiograph (top right) reveals extensive sclerosis in keeping with post radiotherapy osteonecrosis.  Subtle suspicious lucency related to the lateral distal femoral metaphysis.  Radiation induced dystrophic soft tissue calcification also evident. Coronal T1 weighted image (right middle) shows extensive marrow filtration in keeping with biopsy proven radiation induced osteosarcoma. Coronal T1 weighted image from an earlier study 4 years prior (right bottom) confirms extensive intramedullary low signal corresponding to radiation induced osteonecrosis with adjacent preserved intramedullary fat signal at this time (PNG 848 KB)

High Resolution Image (TIF 5.27 MB)

Supplementary Material 6 22-year-old female with a subperiosteal ganglion. Axial PDFS image (left) shows a posterolateral tibial lobular subperiosteal cystic signal structure. Axial T1FS post-contrast image (centre) highlighting slender peripheral enhancement. Axial PDFS image (right) more proximally demonstrates extension to the posterior cortex and towards the proximal tibiofibular articulation (PNG 520 KB)

High Resolution Image (TIF 5.09 MB)

Supplementary Material 7 56-year-old female with chronic ossified subperiosteal haematoma. Lateral knee radiograph (top left) demonstrates anterior ossification extending along the anterior distal femoral metadiaphysis. Sagittal T1 (top right), axial T1 (bottom left) and PDFS (bottom right) images demonstrate corresponding marrow fat signal related to mature subperiosteal ossification with associated remodelling of the supratrochlear femur underlying the patella (PNG 0.97 MB)

High Resolution Image (TIF 4.32 MB)

## Data Availability

Not applicable.
