# Supplementary figures and images for: Intraosseous tumours of the knee
Source: Skeletal Radiol. 2026 Jan 20;55(8):1955–82. doi: 10.1007/s00256-025-05105-y (PMC13309404; doi:10.1007/s00256-025-05105-y)

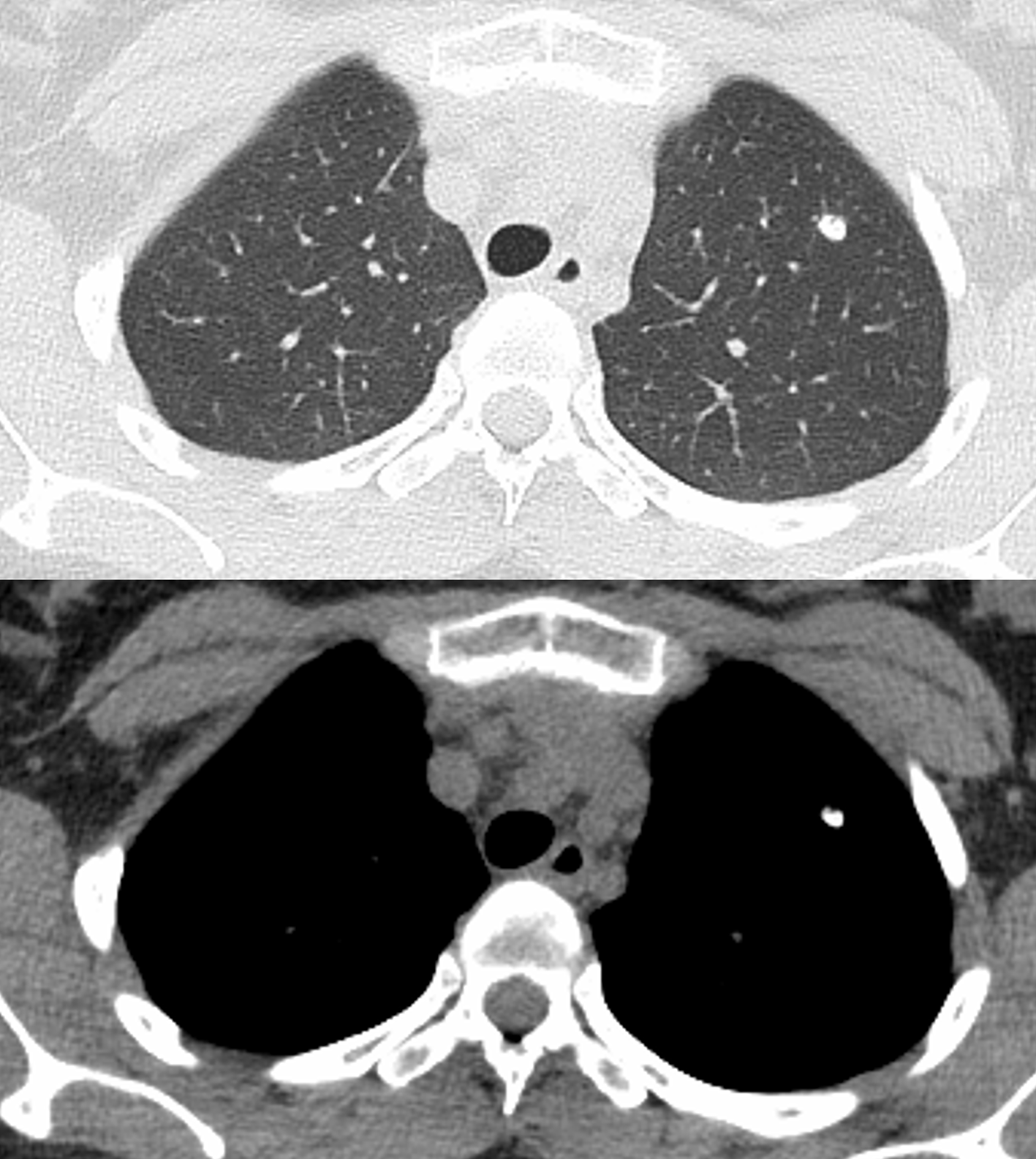

Supplement: Supplementary file 1 — 55-year-old female with metastatic proximal fibular giant cell tumour (Same patient Fig. 3). CT thorax lung (top) and mediastinal (bottom) windows of the same patient show a histologically proven left upper lobe GCT metastasis with associated Denosumab-induced ossification better appreciated on the mediastinal window (PNG 843 KB) [file 256_2025_5105_Fig29_ESM.png]

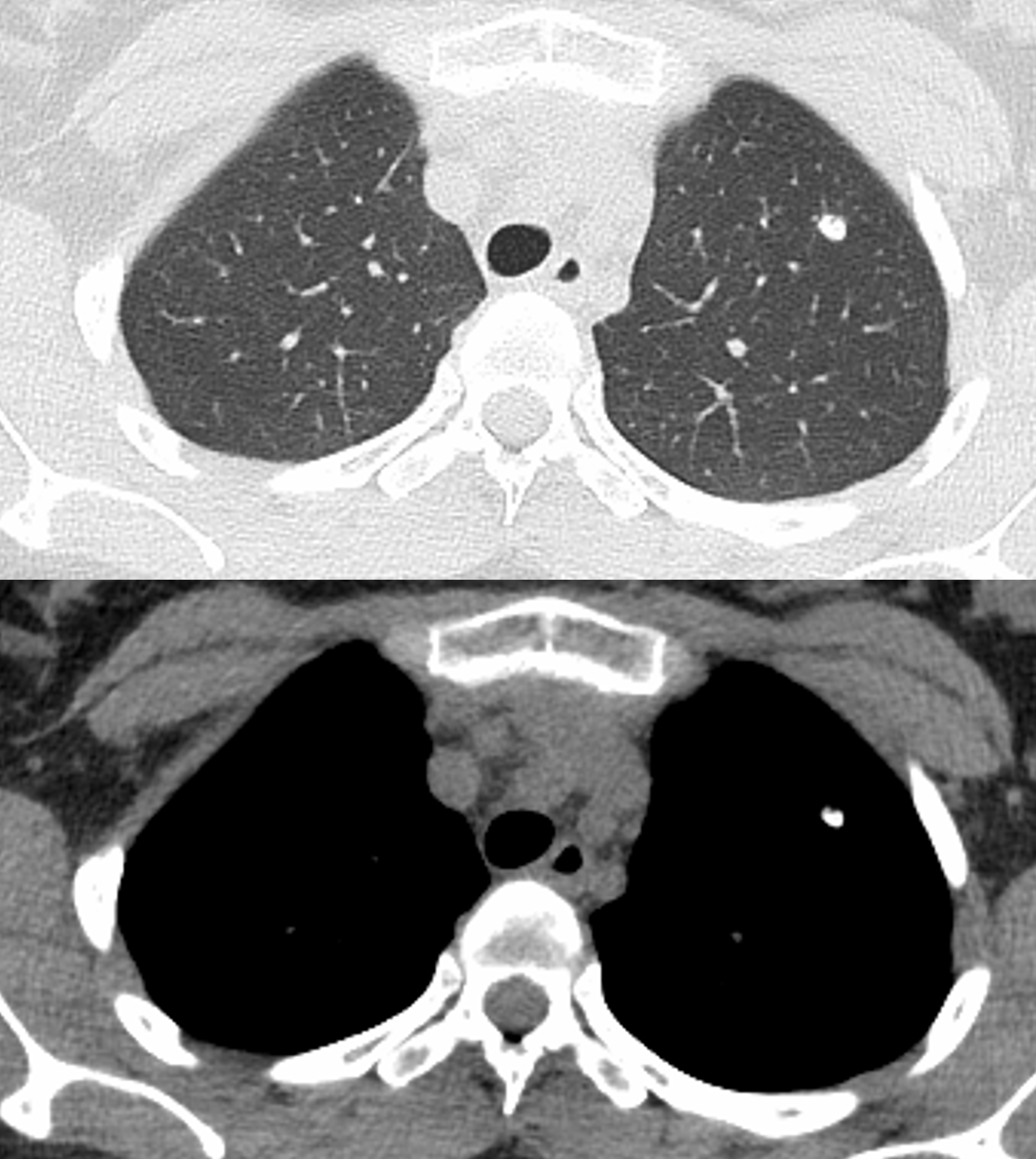

Supplement: Supplementary file 2 — High Resolution Image (TIF 3.71 MB) [file 256_2025_5105_MOESM1_ESM.tif]

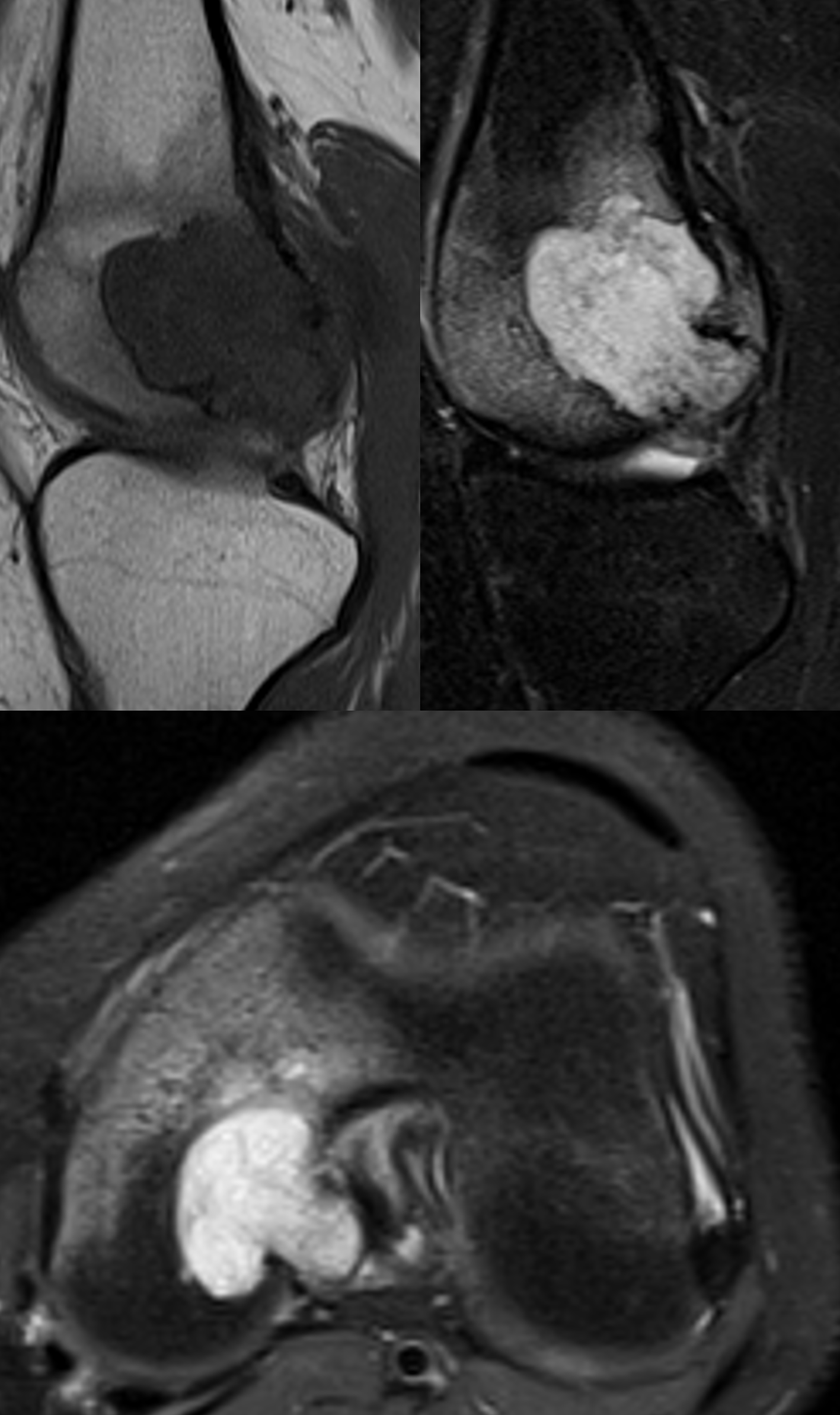

Supplement: Supplementary file 3 — 17-year-old female with central chondrosarcoma Grade 2. Sagittal T1 (top left), STIR (top right) and axial PDFS (bottom) images show a subarticular lesion demonstrating chondral signal characteristics with extraosseous extension into the intercondylar notch and prominent perilesional oedema, which together raise concerns for a high-grade tumour (857 KB) [file 256_2025_5105_Fig30_ESM.png]

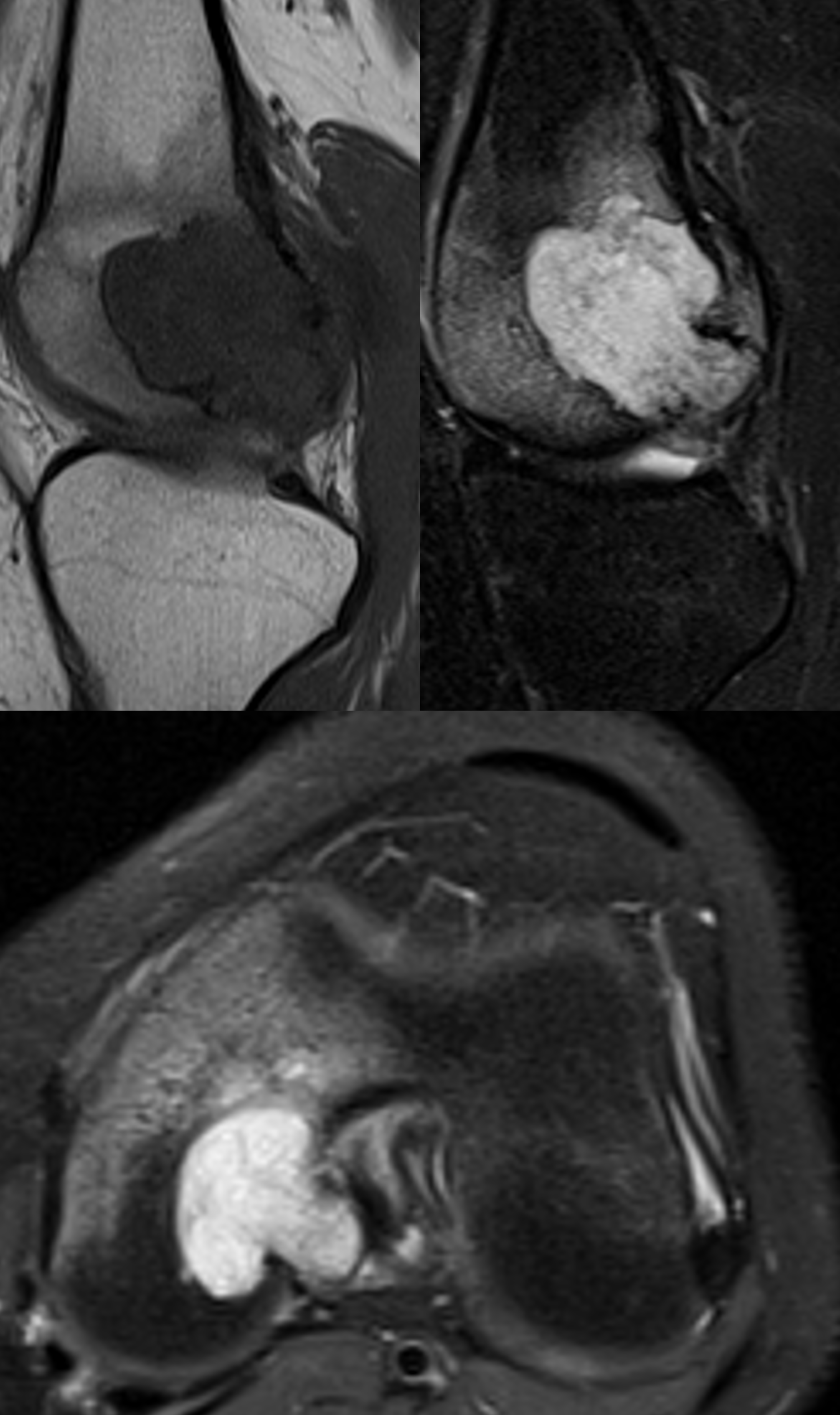

Supplement: Supplementary file 4 — High Resolution Image (TIF 3.79 MB) [file 256_2025_5105_MOESM2_ESM.tif]

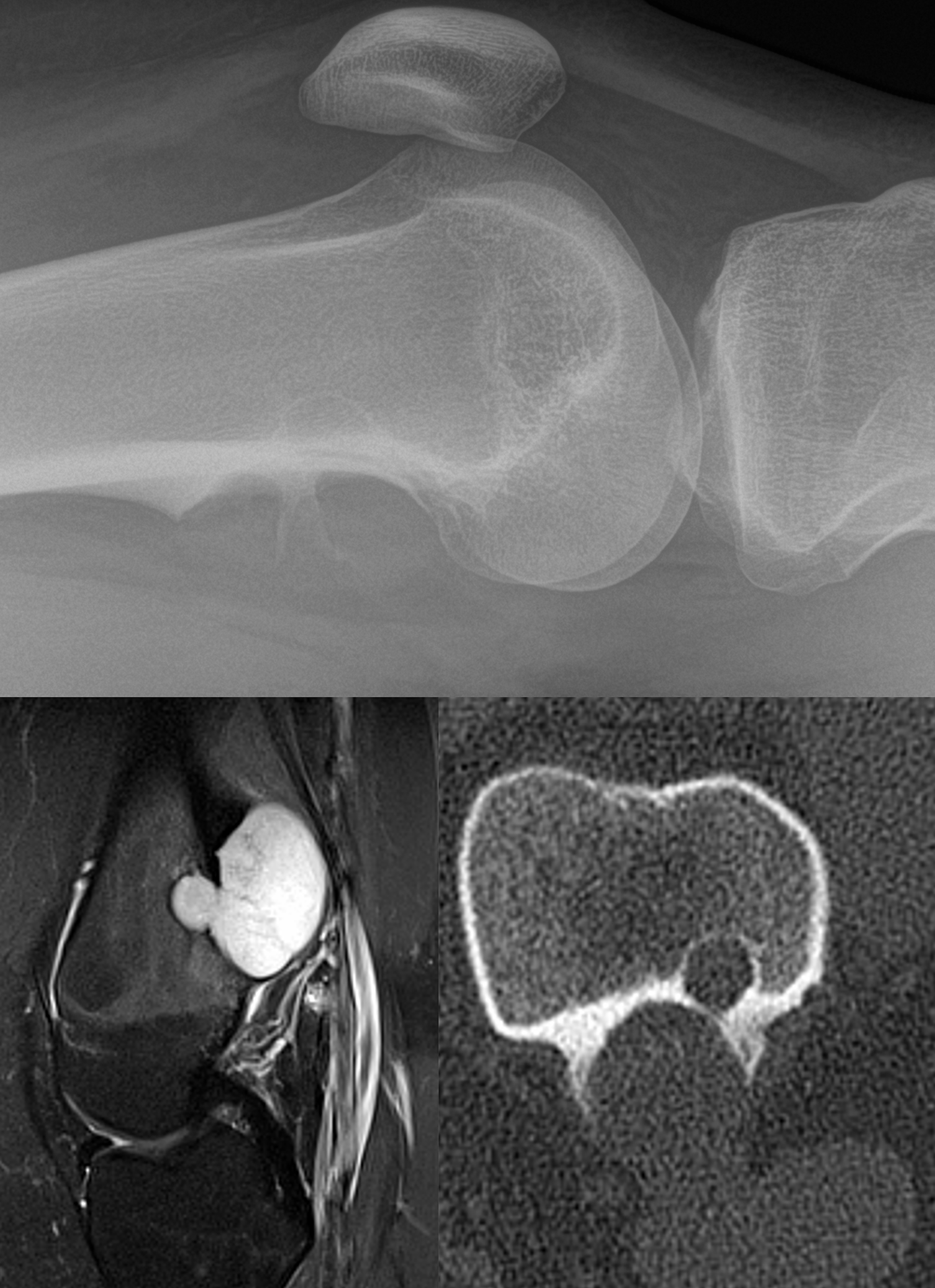

Supplement: Supplementary file 5 — 17-year-old female with periosteal chondroma. Lateral radiograph (top) demonstrates a lucent surface lesion with pressure erosion/cortical scalloping and associated cortical buttress. Sagittal PDFS image (bottom left) reveals a well marginated lobular subperiosteal surface lesion with cortical buttressing and chondral signal characteristics. Intramedullary extension makes differentiation form a chondrosarcoma difficult on imaging but walled off-marginal sclerosis is present. Axial CT (bottom right) confirms cortical erosion with an incomplete periosteal mineralised margin and intraosseous extension with an intramedullary lytic lesion showing peripheral marginal sclerosis (PNG 954 KB) [file 256_2025_5105_Fig31_ESM.png]

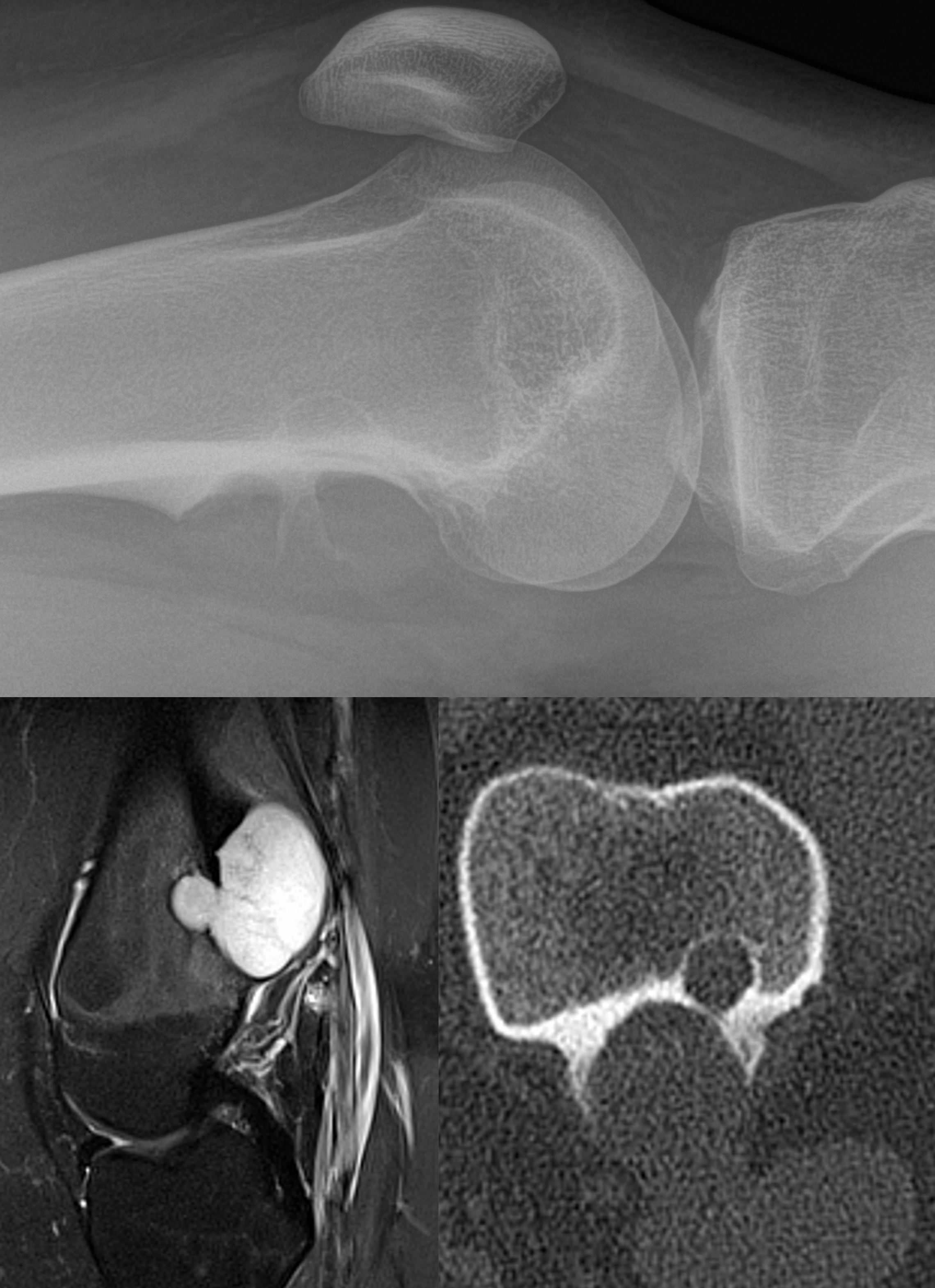

Supplement: Supplementary file 6 — High Resolution Image (TIF 5.24 MB) [file 256_2025_5105_MOESM3_ESM.tif]

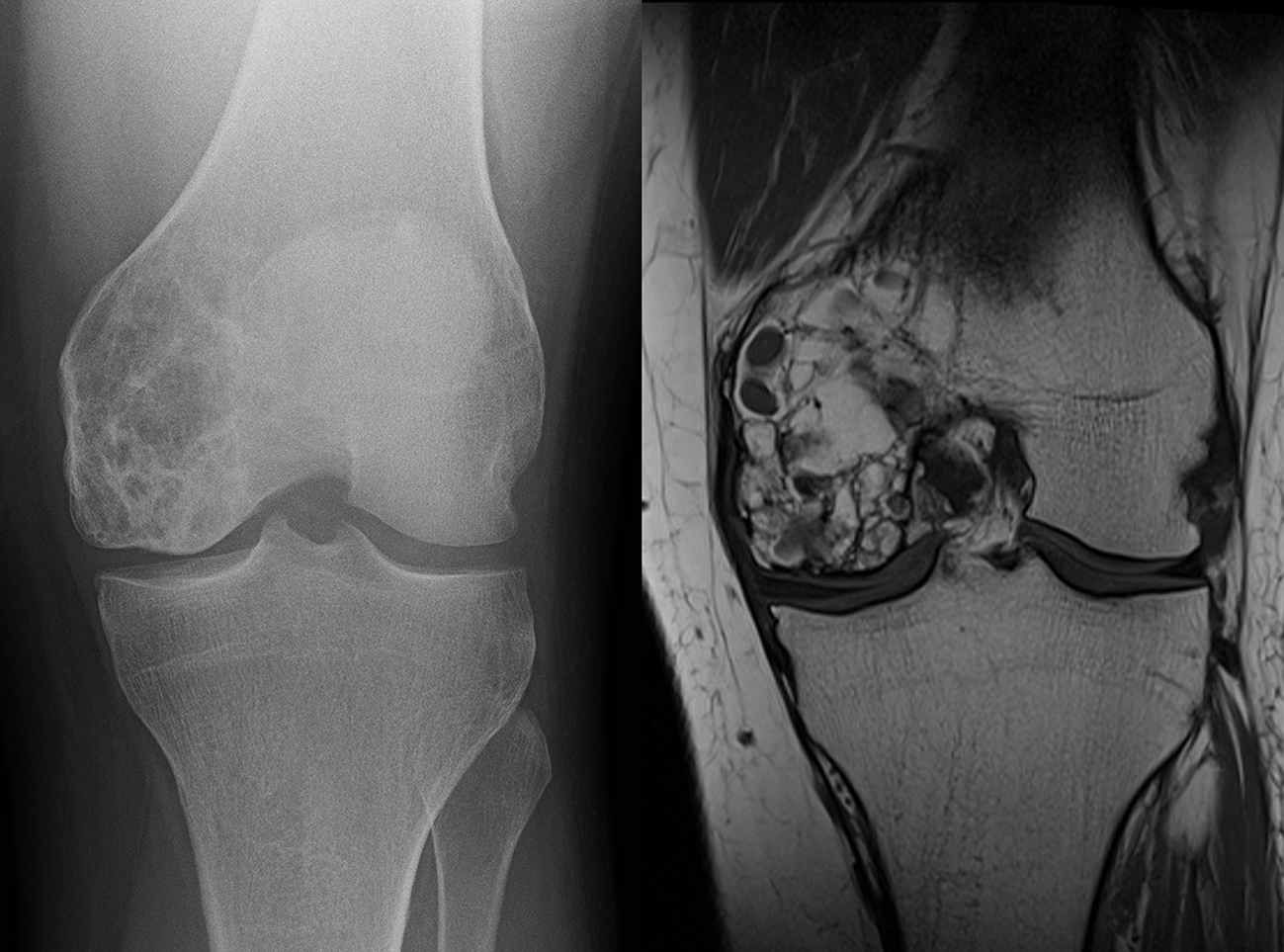

Supplement: Supplementary file 7 — 33-year-old male with intraosseous lipoma. AP radiograph (left) demonstrates a mildly expansile mixed lytic and sclerotic lesion centred within the medial femoral condyle with a peripheral sclerotic margin and internal calcification. Coronal T1 weighted image (right) reveals prominent regions of intralesional fat signal and low signal cystic foci suggestive of fat necrosis (PNG 1.25 MB) [file 256_2025_5105_Fig32_ESM.png]

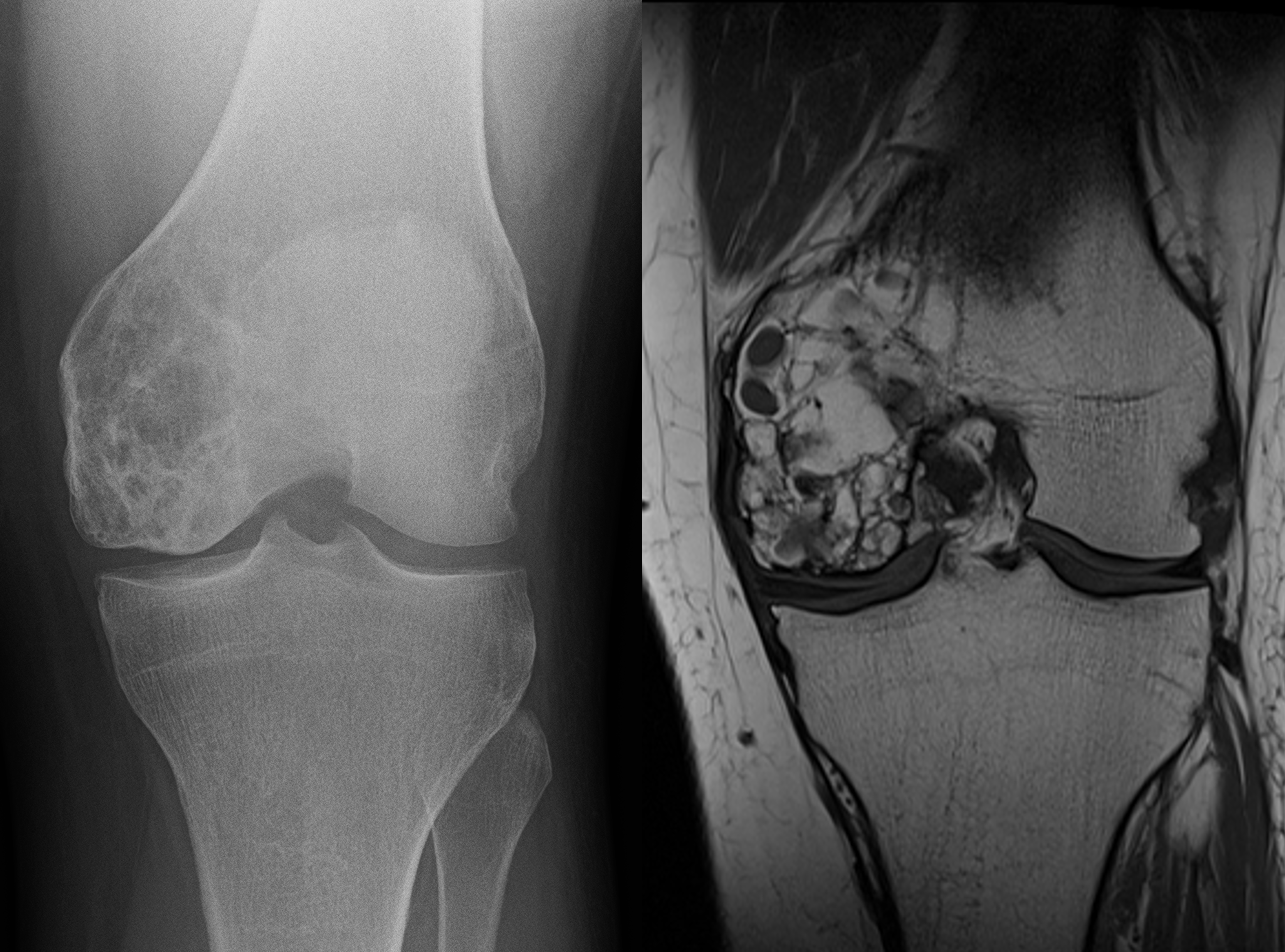

Supplement: Supplementary file 8 — High Resolution Image (TIF 7.02 MB) [file 256_2025_5105_MOESM4_ESM.tif]

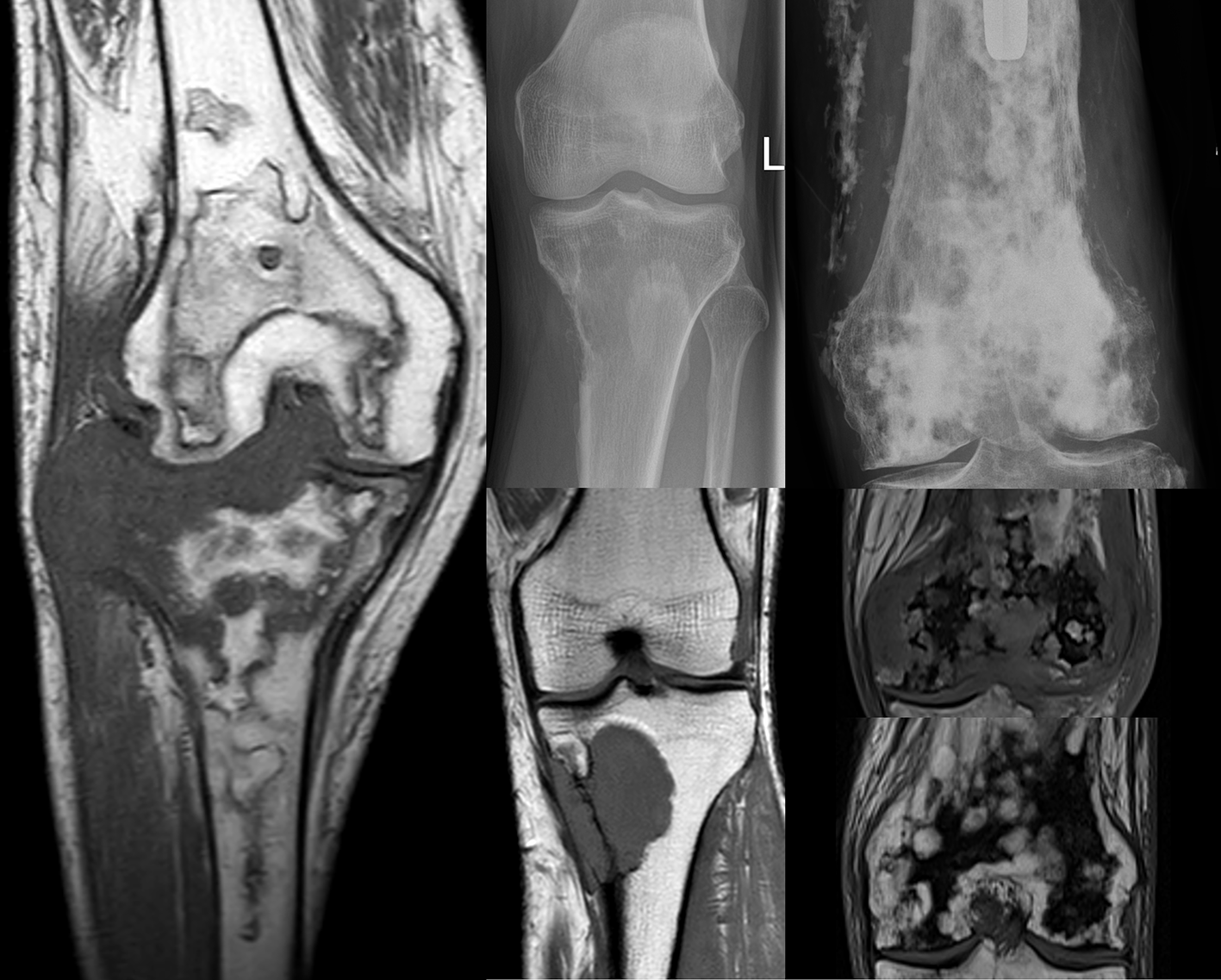

Supplement: Supplementary file 9 — Bone sarcoma. 56-year-old male with infarct associated high grade spindle cell sarcoma (left). Coronal T1 weighted image (left) demonstrates proximal tibial marrow infiltration with extraosseous extension on a background of multifocal osteonecrosis. 50-year-old male with high grade leiomyosarcoma (centre). AP knee radiograph (top centre) shows ill-defined lytic lesion centred on the medial proximal tibial metaphysis, with the superior component demonstrating a well-defined sclerotic margin. Extramedullary mineralisation with cortical erosion suggestive of a subperiosteal component. Prominence of the tibial tuberosity reflects remodelling in keeping with previous Osgood-Schlatter Disease. Coronal T1 weighted image (bottom centre) shows marrow infiltration with the lesion demonstrating regions of differing signal characteristics. Medial subperiosteal component with attenuation of the adjacent cortex. 62-year-old male with radiation induced osteosarcoma. History of previous surgery and radiotherapy for sarcoma 40 years ago with subsequent intramedullary nail for insufficiency fracture (right). AP knee radiograph (top right) reveals extensive sclerosis in keeping with post radiotherapy osteonecrosis. Subtle suspicious lucency related to the lateral distal femoral metaphysis. Radiation induced dystrophic soft tissue calcification also evident. Coronal T1 weighted image (right middle) shows extensive marrow filtration in keeping with biopsy proven radiation induced osteosarcoma. Coronal T1 weighted image from an earlier study 4 years prior (right bottom) confirms extensive intramedullary low signal corresponding to radiation induced osteonecrosis with adjacent preserved intramedullary fat signal at this time (PNG 848 KB) [file 256_2025_5105_Fig33_ESM.png]

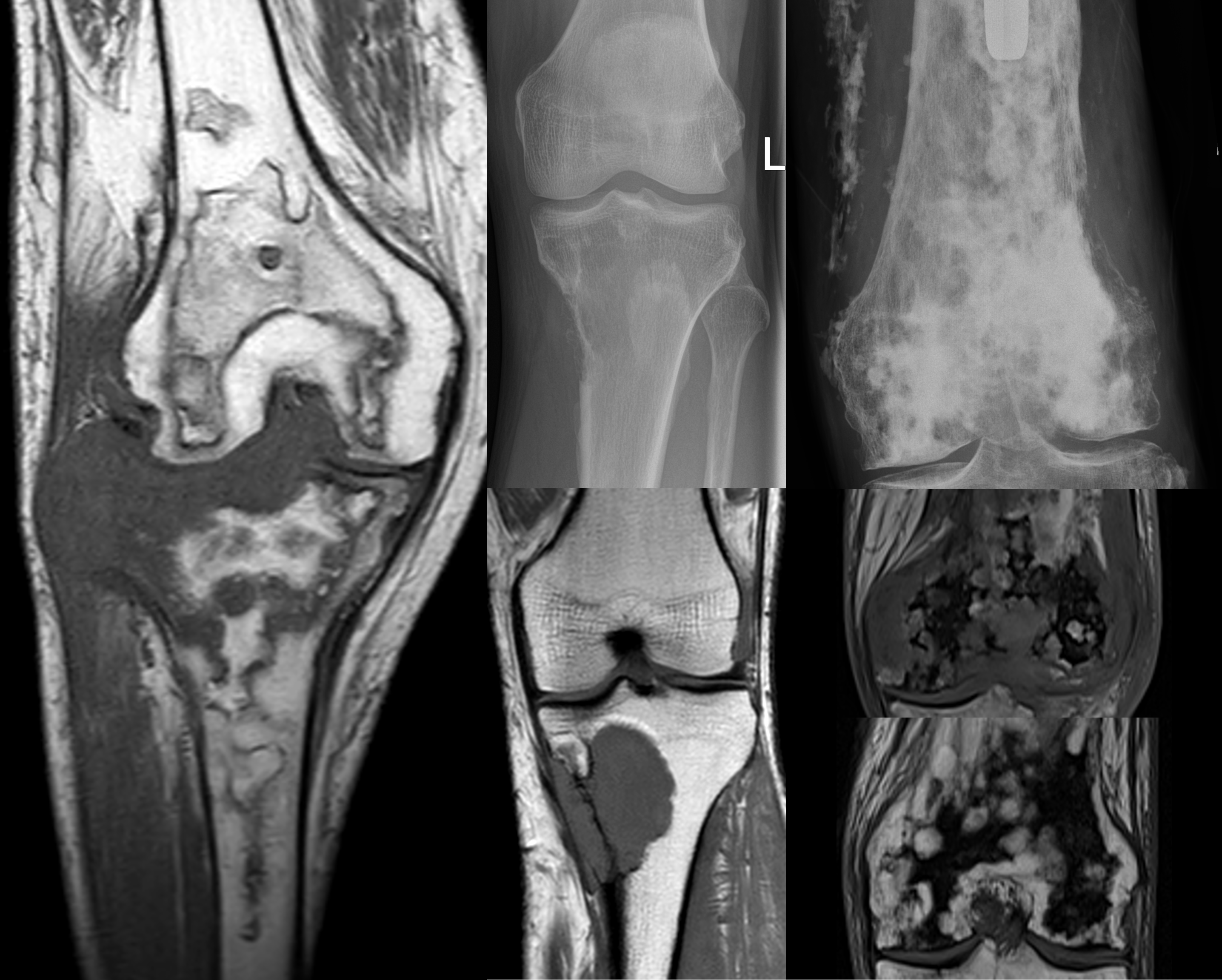

Supplement: Supplementary file 10 — High Resolution Image (TIF 5.27 MB) [file 256_2025_5105_MOESM5_ESM.tif]

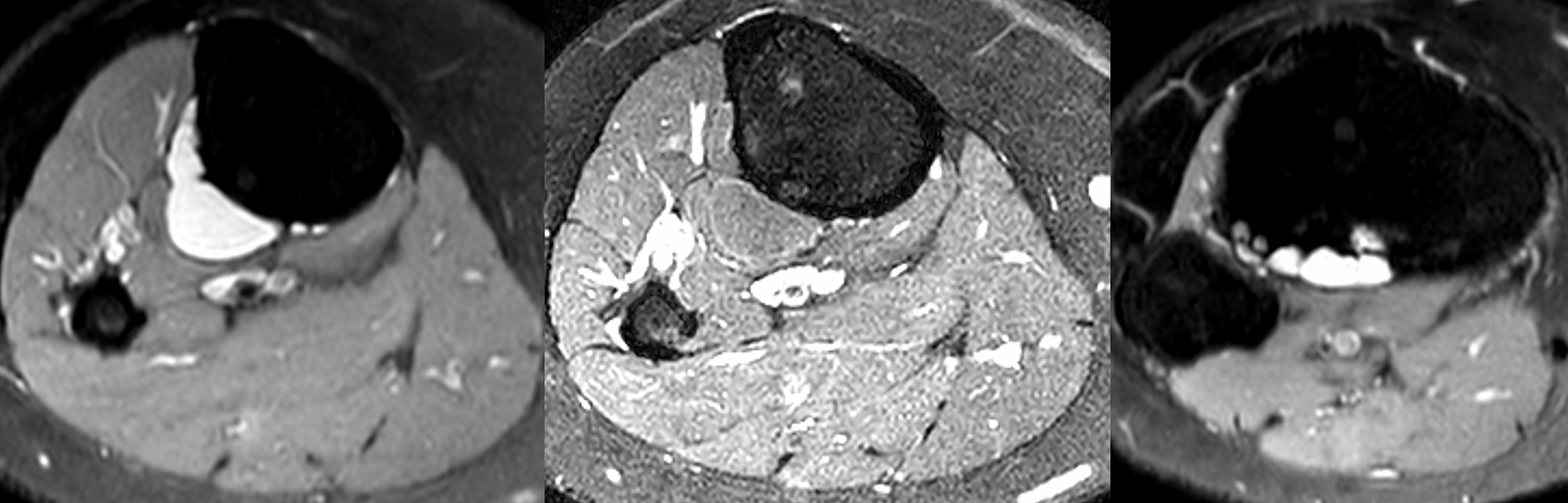

Supplement: Supplementary file 11 — 22-year-old female with a subperiosteal ganglion. Axial PDFS image (left) shows a posterolateral tibial lobular subperiosteal cystic signal structure. Axial T1FS post-contrast image (centre) highlighting slender peripheral enhancement. Axial PDFS image (right) more proximally demonstrates extension to the posterior cortex and towards the proximal tibiofibular articulation (PNG 520 KB) [file 256_2025_5105_Fig34_ESM.png]

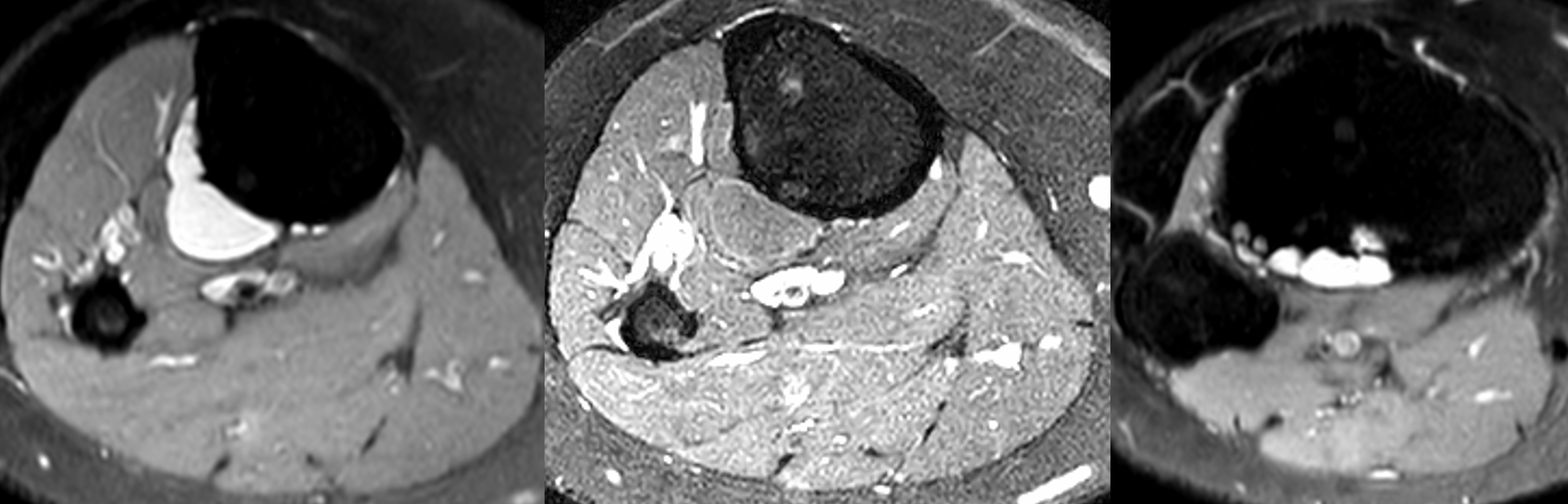

Supplement: Supplementary file 12 — High Resolution Image (TIF 5.09 MB) [file 256_2025_5105_MOESM6_ESM.tif]

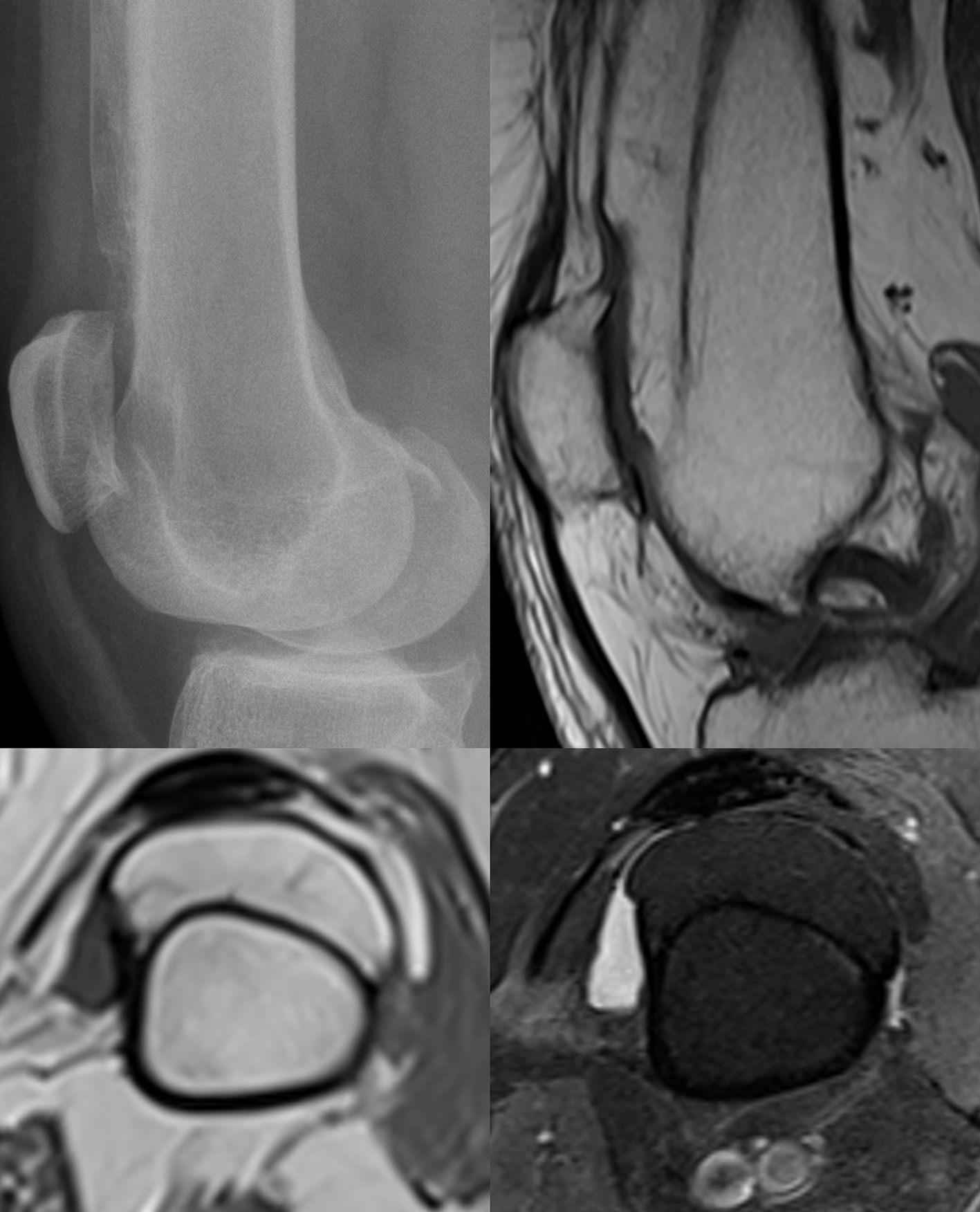

Supplement: Supplementary file 13 — 56-year-old female with chronic ossified subperiosteal haematoma. Lateral knee radiograph (top left) demonstrates anterior ossification extending along the anterior distal femoral metadiaphysis. Sagittal T1 (top right), axial T1 (bottom left) and PDFS (bottom right) images demonstrate corresponding marrow fat signal related to mature subperiosteal ossification with associated remodelling of the supratrochlear femur underlying the patella (PNG 0.97 MB) [file 256_2025_5105_Fig35_ESM.png]

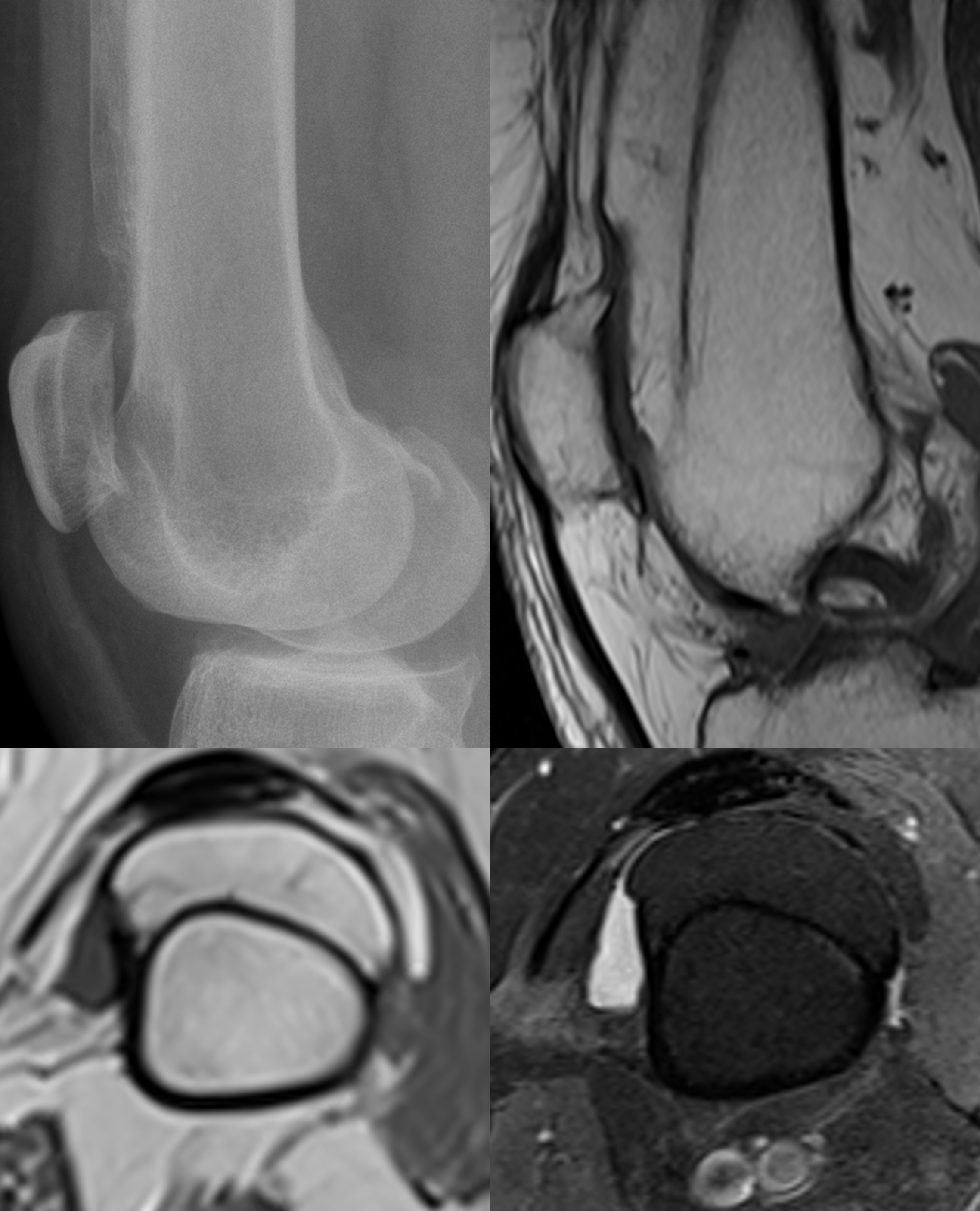

Supplement: Supplementary file 14 — High Resolution Image (TIF 4.32 MB) [file 256_2025_5105_MOESM7_ESM.tif]
